# Instant variable stiffness in cardiovascular catheters based on fiber jamming

**DOI:** 10.1126/sciadv.adn1207

**Published:** 2025-02-07

**Authors:** Yi Sun, Yegor Piskarev, Etienne H. Hofstetter, Cedric Fischer, Quentin Boehler, Zdeněk Stárek, Bradley J. Nelson, Dario Floreano

**Affiliations:** ^1^ Laboratory of Intelligent Systems, École Polytechnique Fédérale de Lausanne, 1015 Lausanne, Switzerland.; ^2^ Multi-Scale Robotics Laboratory, Eidgenössische Technische Hochschule Zürich, 8092 Zürich, Switzerland.; ^3^ 1st Department of Internal Medicine, Cardioangiology, St. Anne’s University Hospital in Brno and Faculty of Medicine of Masaryk University, Brno, Czech Republic.; ^4^ Interventional Cardiac Electrophysiology Group, International Clinical Research Center, St. Anne’s University Hospital Brno, Pekařská 53, 602 00 Brno, Czech Republic.

## Abstract

Variable stiffness (VS) has revolutionized miniature surgical instruments, including cardiovascular catheters for minimally invasive surgeries (MISs), enabling advanced capabilities in stiffness modulation and multi-curvature bending. However, existing VS catheters with phase-changing materials are slow in softening and stiffening rates (≈90 s), which can lead to substantial increase in surgery duration. To address the slow stiffness change, we propose a VS catheter based on fiber jamming (FJ) that achieves instant stiffness changes (≤300 ms), enabling seamless catheter operations without delays. Moreover, our catheter, incorporating hundreds of ultrathin fibers into a slender 2.3-mm catheter body, achieves up to 6.5-fold stiffness changes. With adequate stiffness change, our two-segment catheter achieves complex bending profiles within seconds. In addition, the FJ-based design does not require electric currents or heating inside the human body, minimizing patient risks. This FJ-based VS catheter, with instantaneous response, adequate stiffness change, and enhanced safety, can potentially establish benchmarks in MIS, allowing medical practitioners to effectively address formidable diseases.

## Teaser

Fiber jamming catheter achieves variable stiffness with instant response, adequate stiffness change for cardiovascular surgeries.

## INTRODUCTION

Minimally invasive surgery (MIS), such as cardiac ablation for treating arrhythmia, aims to deliver safer procedures with minimized lesions, reduced infection risks, improved surgical outcomes, and shorter recovery times (*1*–*4*). Miniature surgical instruments with dexterous motion capabilities, called catheters, are used in MISs. Such a surgery can be performed either manually or remotely using a remote magnetic navigation (RMN) system. This system equipped with a magnetic catheter generates a controllable magnetic field using multiple electro-magnets surrounding the patient body to steer the magnetic catheter inside the patient during surgical intervention (*5*, *6*). This allows the motion mechanism to be separated from the catheter, miniaturizing the catheters with simpler structures, enabling remote surgery execution by the doctors without exposure to x-rays during fluoroscopy, and reducing the training burden on doctors (*5*, *6*). However, existing magnetic catheters can achieve only uni-curvature bending, limiting their workspace and dexterity. Therefore, many surgical regions involved in cardiac ablation are challenging to reach by the existing catheters due to the complex anatomy of the human heart (*7*–*10*).

To improve the dexterity, researchers have developed multi-segment catheters with variable stiffness (VS) capability (*11*–*14*). These catheters are composed of a few segments made using phase-changing materials whose stiffness can be reversibly changed from rigid to soft state by engaging and disengaging a thermal stimuli (*11*–*14*). Therefore, their segments can be selectively manipulated with sequential stiffening and softening of the corresponding segments to form complex multi-curvature shapes, thereby increasing the reachable and dexterous workspace of the catheters (*11*–*14*). Several phase-changing materials with low melting points have been used in the VS catheters such as the low-melting-point alloy and shape memory polymers, and they have achieved stiffness change factors (SCFs) (the stiffness ratio of the stiff state to the soft state) of at least 20 (*11*–*14*).

However, despite the high SCFs of these devices, their complete stiffness change cycle from the rigid to soft state and vice versa can take up to 90 s due to the slow heating and cooling rates of the materials (table S1) (*11*–*14*). As a result, the doctor must wait frequently for the next manipulation of the catheter, which substantially prolongs the surgical procedure. In the case of cardiac ablation surgery, which usually involves multiple ablation sites and dozens of ablation points per site, the catheter must be re-positioned many times (*15*–*17*). With repeated slow heating and cooling processes, the accumulative waiting time can extend to hours when such VS catheters with phase-changing materials are used, compromising the surgical efficiency, increasing the costs, and limiting patient access to treatments. In addition, the use of electric power for heating inside the human body imposes a heavy concern for patient safety.

**Fig. 1.**
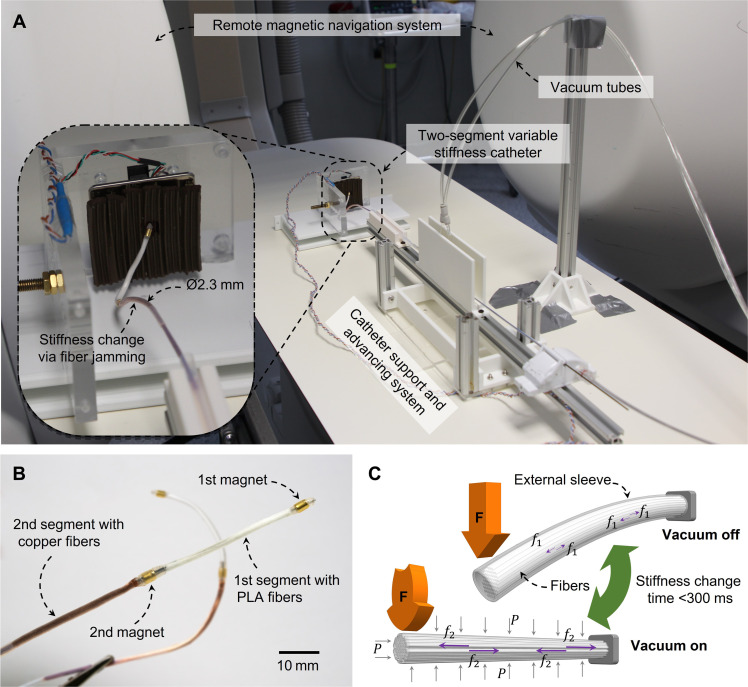
Overview of the FJ VS catheter. (**A**) Two-segment catheter in the clinical setting. The catheter support and advancing system moves the catheter back and forth, the RMN system manipulates the tip of the catheter, and the vacuum tubes regulate the catheter stiffness. (**B**) Catheter prototypes with two FJ segments and magnets. (**C**) FJ working principle. The vertical orange arrows represent the external force to bend the FJ beam; the horizontal purple arrows represent the frictional forces (*f*) between the fibers when the vacuum is off (*f*_1_) and on (*f*_2_), where *f*_1_ << *f*_2_; the gray vertical arrows represent the external pressure (*P*) applied to the external sleeve upon vacuum application.

Here, we present a VS catheter based on fiber jamming (FJ) that can undergo rapid stiffness changes within 0.3 s. This catheter consists of two VS segments filled with fibers of different materials [polylactide acid (PLA) and copper fibers] and two magnets to manipulate the two segments via a controllable magnetic field from a hospital-compatible RMN system (Fig. 1, A and B). Upon selective vacuum application, one segment can be stiffened, while the other segment remains soft for manipulation. In our FJ configuration, the copper segment has a higher stiffness range, whose stiff state provides strong anchoring for the manipulation of the distal PLA segment, which has a lower stiffness range. When the catheter forms the desired curve, the two segments can be locked in shape upon vacuum application for the ablation procedure to proceed (fig. S1). The stiffness change processes require only the on-off switches of the vacuum application with instant stiffness change in the catheter, making this process substantially faster than that of the existing VS catheters that rely on phase-changing mechanisms.

FJ devices comprise a slender sleeve containing numerous fibers that can freely move inside the sleeve (*18*–*21*) (Fig. 1C). When air is evacuated from the sleeve via vacuum application, the external pressure squeezes the fiber bundle and increases the mutual friction of the fibers. This increased friction impedes the relative sliding of the fibers, resulting in an increase in the bending stiffness (*18*–*20*). FJ technology has recently emerged as a promising VS mechanism with potentials in different robotic applications, such as robotic manipulators, grippers, and wearable devices (*19*, *22*–*26*). In particular, FJ is suitable for surgical instruments with tubular structures that require omnidirectional bending stiffness changes (*19*, *21*). Recent studies have reported FJ-based devices with jamming ratios (equivalent to SCF) up to 20 by exploring different materials, roughness, shapes, and arrangements of the fibers (*18*, *20*, *25*, *27*). However, the existing FJ prototypes are 10 times larger than the size of the cardiovascular catheters, which typically have diameters of 2.33 mm. This dimension requirement imposes formidable challenges in catheter fabrication with FJ integration to achieve desirable SCF performance.

To make the catheter body ultra-slim, packing conventional fibers with circular cross sections in a soft sleeve, as opposed to those with square cross sections or rough surfaces, remains feasible. However, the SCF of such FJ configuration is below 3 (*18*, *20*), which is not sufficient for the cardiovascular catheter applications as they require an SCF of at least 4 (section S1). To overcome this challenge, our strategy for achieving a high SCF is to incorporate as many thin fibers as possible (section S2). The existing FJ-based devices are filled with less than 100 fibers with diameters of at least 500 μm in a thicker sleeve (*19*, *22*–*26*). Therefore, the existing fabrication approaches do not enable manufacturing a large number of ultrathin fibers and packaging them in the scale of Ø2.33 mm suitable for cardiac catheters.

To address this challenge, we introduce an innovative fabrication procedure to construct the FJ-based VS catheter. The proposed procedure includes a precise method to produce fibers as thin as 50 μm and a process to pack up to 1000 fibers within a 2-mm-diameter space. With our fabrication, we conducted parametric optimization experiments on FJ and obtained the optimal SCFs up to 6.5, possibly the maximum SCF achievable for FJ at the catheter scale. To tune the catheter stiffness range for the magnetic field magnitude, which differs for various surgery applications, we also explored the use of hybrid fiber bundles consisting of PLA and copper fibers with different material ratios. Furthermore, we created a method to measure the stiffness change time of FJ, which has not yet been achieved in the previous articles. The measurements show that the FJ stiffness changes occur within 300 ms, which is two orders of magnitude faster than the change rate of the existing VS catheters. Tests under the RMN system show that the single-segment FJ catheters can bend smoothly in the soft state upon the application of a magnetic field, and maintain minimal deflection in the rigid state when the fibers are jammed. A two-segment catheter is then fabricated and tested with the RMN system, which allows selective bending to achieve two-curvature profiles and manipulation inside a three-dimensional (3D) phantom of the human heart. Compared to the existing VS catheters, the proposed FJ catheter achieves adequate and ultra-fast stiffness changes with safe materials and simple control. In addition, it eliminates the need for heating or electric power working inside the human body. Furthermore, our fabrication can be adopted to produce other FJ catheters for different specific MIS procedures. Therefore, our proposed FJ-based VS catheter has strong potential to refine MIS by reducing procedure time and costs, and enhancing safety for both patients and surgeons.

## RESULTS

### Design and fabrication of the catheter

The FJ VS catheter has two segments, connected by a long polytetrafluoroethylene (PTFE; for the material acronyms, please refer to table S2) tube to the rear tubing assembly (figs. S2 and S3A). The two segments have similar structures with a magnet at the tip of each segment followed by the fibers (fig. S2). They are encapsulated inside a silicone sleeve with silicone glue at the tip to fix the fibers and seal the segments (figs. S2 and S3, B and C). In addition, one tube (purple) serving as the working channel is placed through the two segments for future installation of the ablation tip, and another tube (green) is positioned up to the first/PLA segment for vacuum application (figs. S2 and S3, C and D). The vacuum application for the second/copper segment is served through the long PTFE tube (figs. S2 and S3D). At the rear tubing, the PTFE tube, the working, and vacuum channels are decoupled into three independent outlets (fig. S3, A and E). Between the two segments, there is a supporting carbon fiber (CF) rod extending from the tip of the second segment into the PLA fibers of the first segment to serve as a stiff support (figs. S2 and S3C). Detailed component information is given in fig. S2 and table S2, while the anatomy of the catheter with its true proportions is illustrated in fig. S3.

**Fig. 2.**
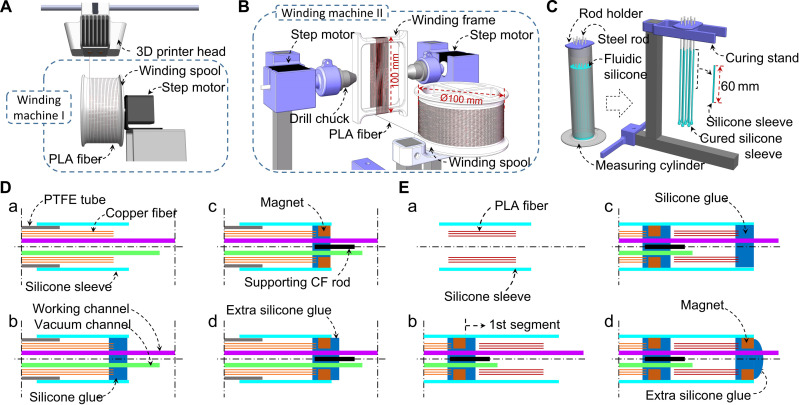
Fabrication of the FJ VS catheter. (**A**) Ultrathin PLA fibers are produced by winding machine I, which pulls the melted PLA filament through its spinning spool. (**B**) Fiber bundles with specific numbers of fibers are prepared on winding machine II. (**C**) Silicone sleeves are fabricated by dipping steel rods (Φ2 mm) in the freshly mixed silicone fluid and then by curing them on the curing stand. (**D**) Fabrication of the second FJ segment. The silicone sleeve is first inserted into the right side of the PTFE tube, followed by the insertion of the copper fibers and the working and vacuum channels [(D), a]. Silicone glue is then applied at the right tip to fix the copper fibers [(D), b], followed by the immediate insertion of the magnet and the supporting CF rod [(D), c]. Extra silicone glue is then applied to seal the second segment [(D), d]. (**E**) Fabrication of the first FJ segment. The PLA fiber bundle is first inserted into the silicone sleeve [(E), a]. The halfway first segment is inserted onto the tip of the second segment along the working channel. The silicone sleeve is then rubbed leftward and fixed at the tip of the second segment with silicone glue [(E), b]. The right end of the PLA fibers is fixed with silicone glue [(E), c], followed by the immediate insertion of the magnet and sealing with extra silicone glue [(E), d].

To fabricate the catheter, we first obtain a large quantity of ultrathin fibers of two material types. We select enameled copper wires (Ø50, 75, 100 μm) and PLA fibers (Ø50 μm) extracted from a 3D printer (Ultimaker S5, Ultimaker B.V.) using winding machine I to pull the heated PLA filament (Fig. 2A, section S3, and movie S1). Once the fibers are prepared, the winding spool will be mounted onto winding machine II, which reformats the fibers into a fiber bundle (Fig. 2B). By applying specific numbers of rotations of the winding frame, a bundle with the desired number of fibers is obtained. The second step involves preparing the external sleeve for packing the fiber bundle. We choose silicone rubber (DragonSkin 0020, Smooth-On Inc.; elastic modulus at 100% strain: 0.34 MPa), which, in the form of thin skin, is neither too brittle to break during manipulation nor too stiff to affect the jamming effect. A dipping-curing process is used to form silicone sleeves with a consistent wall thickness (≈150 μm) (Fig. 2C).

With all the components ready, the catheter fabrication proceeds with copper fiber segment construction (Fig. 2D) followed by the PLA fiber segment (Fig. 2E). Both of the segments are fabricated similarly, involving the insertion of the fibers into the silicone sleeve, the insertion of the working and vacuum channels, the glue application, and the magnet insertion. The main difference of the two-segment fabrication lies with the supporting CF rod at the tip of the second segment (Fig. 2Dc) and the location of the vacuum channel tip, which stays in the first segment (Fig. 2Eb). The whole fabrication process is elaborated in Materials and Methods and movie S1. Some key relative positions for the catheter fabrication process are provided in fig. S4, which illustrates the components with their true proportions. For the rear tubing assembly and its functionality, please refer to fig. S5.

### FJ characterization and optimization

To investigate FJ performance in terms of stiffness variation at the catheter scale and determine the optimal FJ configuration, we conducted detailed characterization using the three-point bending tests by varying different parameters: filling rate (the ratio of the sum of cross-sectional area of all fibers to the inner cross-sectional area of the silicone sleeve), fiber diameter, and fiber material. Moreover, the FJ reaction times are also measured via our method. With the focus on the FJ performance, all characterizations are implemented using the single-segment catheters with 40-mm-long FJ segments (fig. S6).

**Fig. 3.**
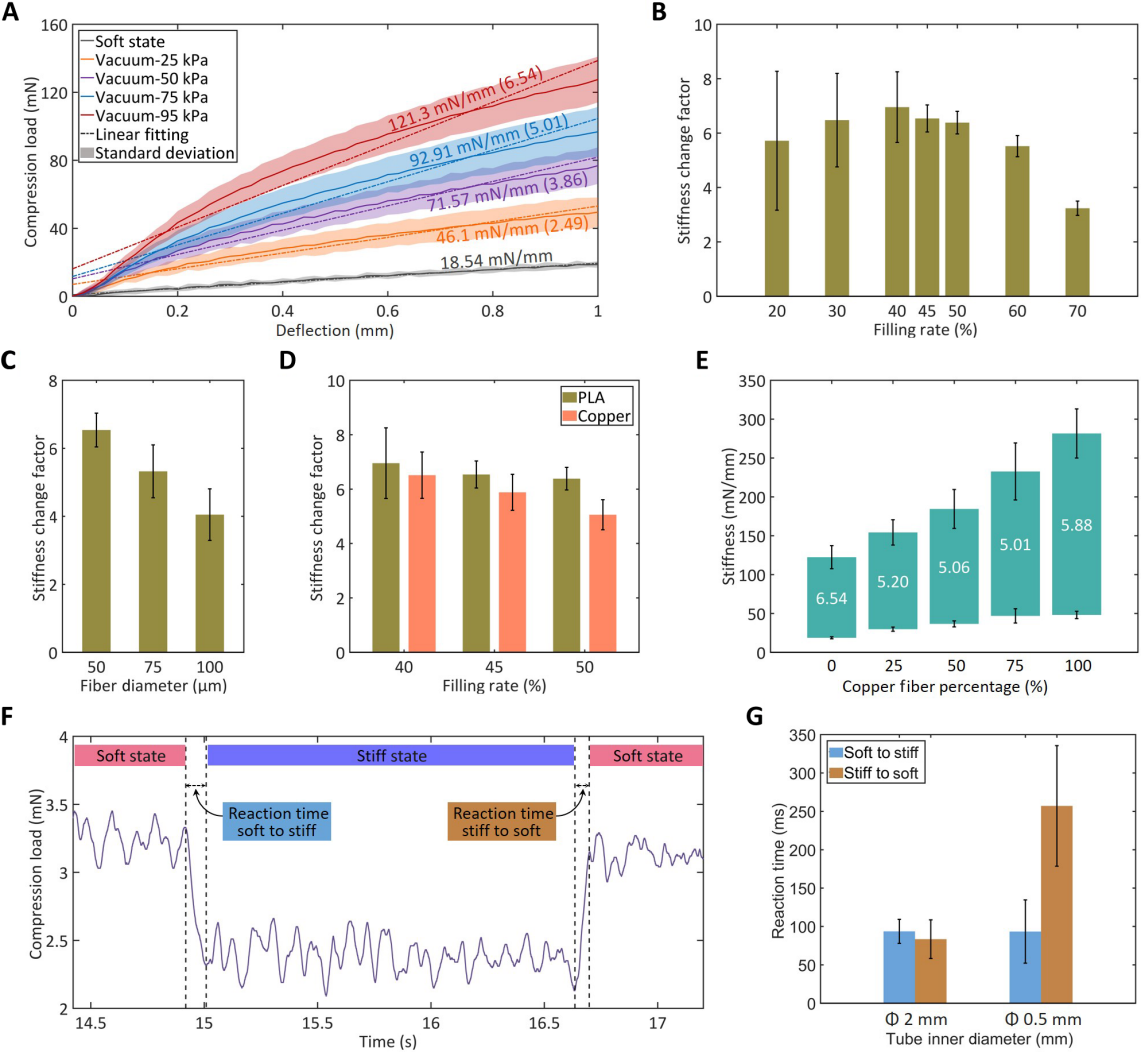
Stiffness and reaction time characterization of FJ at catheter scale. (**A**) Force-deflection curves of the FJ segments with 45% filling rate and Ø50-μm PLA fibers at different vacuum levels. The slopes of the individual linear fitting lines, representing the stiffness, are annotated with matching colored values. The SCF values are in parentheses. The force curves with vacuum application are nonlinear, featuring steeper slopes in the initial deflection range (0 to 0.2 mm) and gentler slopes in the medium deflection range (0.2 to 1 mm). (**B**) SCFs of FJ with Ø50-μm PLA fibers at different filling rates. (**C**) SCFs of FJ with PLA fibers of different diameters at 45% filling rate. To reach 45% filling rate, the numbers of fibers with 50, 75, and 100 μm diameters are 720, 320, and 180, respectively. (**D**) SCFs of FJ with Ø50-μm copper and PLA fibers at the optimal filling rates. (**E**) Stiffness and SCFs (white numbers) of FJ at 45% filling rate using the hybrid Ø50-μm fiber bundles with different PLA-copper compositions. (**F**) Typical force profile of the FJ transition from soft to stiff and from stiff to soft states (FJ sample: 45% filling rate with Ø50-μm PLA fibers). High and low force levels represent the soft and stiff states of the FJ, respectively. Transition times between the two force levels indicate FJ reaction times. (**G**) FJ reaction time measured from the catheters with Ø50-μm PLA fibers at 45% filling rate using 2-m-long tubes with 2- and 0.5-mm IDs to connect the vacuum source. In (B) to (G), all results are obtained using a −95-kPa vacuum. All error bars represent one SD.

Before the investigation of the design parameters, the force profiles are obtained, by means of three-point bending tests (fig. S7), to analyze the FJ behavior of a catheter with PLA fibers, a filling rate of 45%, and a diameter of Ø50 μm (Fig. 3A). Stiffness and SCF rise with increasing vacuum pressure, revealing the possibility of continuously modulating the catheter stiffness. The SCF meets the requirement (at least 4) for the catheter application with a vacuum below −75 kPa.

In the force curves with vacuum application, the steep slope in the initial deflection range (0- to 0.2-mm deflection) represents the high stiffness of the fiber bundle, with the static friction preventing the relative sliding of the fibers. However, this deflection range is too narrow for practical applications. The medium deflection range (0.2- to 1-mm deflection) yields lower stiffness due to the lower kinetic friction of the fiber sliding after overcoming the static friction.

According to the previous articles (*18*–*21*), a more practical way to represent the stiffness of the FJ segment in the stiff state is to use the combined stiffness (a stiffness that combines higher stiffness in the initial deflection of the FJ segment with the lower stiffness in the medium range). In our case, we calculate the combined stiffness using linear fitting of the force curves across the initial and medium deflection range (0- to 1-mm deflection) (Fig. 3A).

The stiffness and SCF for both 0- to 0.2-mm and 0.2- to 1-mm deflection ranges are also calculated using linear fitting (table S3). In the medium deflection range, our catheter also meets the SCF requirement of SCF at −75-kPa vacuum. Meanwhile, SCF at the 0- to 0.2-mm deflection range can reach as high as 12.2 when maximum vacuum is applied.

The first parameter investigated is the filling rate with Ø50-μm PLA fibers at maximum vacuum (−95 kPa). As the filling rate is increased from 20%, the average SCF initially increases, peaks at a filling rate of approximately 40%, and then drops (Fig. 3B). The standard deviations (SDs) of the SCFs are greater than 1 at low filling rates, representing a poor repeatability of the VS performance of the catheter (Fig. 3B). At the low filling rates, the FJ segment is somewhat hollow and, upon vacuum application, the cylindrical FJ segment is squeezed into a flattened body (fig. S8, A and B). Consequently, testing the FJ segments at different orientations (fig. S7B) results in SCFs that can range from 3.6 to 8.2 (Fig. 3B). As the filling rate is increased to more than 45%, the SD drops below 0.75, representing consistent FJ performance (Fig. 3B). At high filling rates, the FJ segment becomes more densely packed with the fibers, and, therefore, its cross section remains circular after vacuum application (fig. S8, C and D), which yields similar SCFs at different orientations (Fig. 3B). However, the higher filling rates come with higher stiffness in the soft state due to the increased initial friction caused by fiber congestion (fig. S9A), resulting in lower SCFs (Fig. 3B). By comparison, the best filling rate for the catheter is determined to be 45%, as this value leads to a relatively high SCF (6.54) and low SD (0.5).

With the optimal filling rate at 45%, the impact of fiber diameter on stiffness is then investigated with PLA fibers at −95-kPa vacuum. The results show that increasing fiber diameters from 50 to 100 μm reduces the SCF from 6.5 to 4 (Fig. 3C). The decrease in the SCFs is primarily due to the reduced stiffness in the stiff state as the fiber diameter increases (fig. S9B). At the same filling rate, fewer fibers can be included in the sleeve when using thicker fibers. The reduced number of fibers leads to fewer contact points between the fibers, resulting in reduced friction and thus lower bending stiffness in the stiff state. Meanwhile, using thicker fibers slightly increases the stiffness of the catheters in the soft states (fig. S9B), which also contributes to the low SCFs. Therefore, Ø50 μm is chosen as the optimal fiber diameter for the catheter.

Copper fibers are also tested at the best three filling rates using −95-kPa vacuum. The SCF and SD of the catheters with copper fibers also decrease with increasing filling rate (Fig. 3D). Because of the smoother surface, the copper fibers exhibit SCFs 0.2, 0.66, and 1.32 lower than those of the PLA fibers at 40, 45, and 50% filling rates, respectively. However, the absolute stiffness of the copper fiber catheter in the stiff state is approximately double that of the PLA fiber catheter (fig. S9C). Thus, the copper fiber is a potential option for making high-stiffness catheters. On the basis of these experiments optimizing the three variables, the best FJ configuration is determined to be a filling rate of 45% with 50-μm fiber diameter, applicable to both PLA and copper fibers.

The utilization of the two materials (PLA and copper) with different stiffness provides the possibility to customize catheter stiffness by mixing them with different ratios into hybrid fiber bundles. Therefore, hybrid fiber bundles with 75 to 25%, 50 to 50%, and 25 to 75% PLA-copper ratios are prepared (fig. S10) and tested. The stiffness ranges of the hybrid fiber bundles gradually shifted from that of a 100% PLA bundle to that of a 100% copper bundle as the percentage of copper fibers increases and the percentage of PLA fibers decreases (Fig. 3E). However, the SCFs of the hybrid bundles remain stable, averaged at 5.1, as the fiber ratio changes. This stiffness customization allows us to configure the stiffness of the multi-segment catheters, as explained in section S4. The values of the measured stiffness of the catheter vary depending on the aforementioned factors, but they are inside the theoretical range according to our simplified model (section S5).

FJ technology is known for its fast reaction upon vacuum application. However, the actual reaction time (time required for a complete stiffness change from the soft to the stiff state or vice versa) has not been previously reported due to the lack of a precise measurement method. Here, we devised a method to measure the FJ reaction time (see Materials and Methods). We first obtain the force profile during vacuum switching, which yields different force levels in the vacuumed/rigid and nonvacuumed/soft states (Fig. 3F). By measuring the transition times between the two force levels, the reaction (stiffening and softening) times of FJ are obtained (Fig. 3F). Vacuum tubes of different diameters (2 and 0.5 mm for the PTFE tube and the vacuum channel, respectively) with 2-m length are attached to the catheters for testing. The results show that the reaction times are mostly below 100 ms, except for the softening times with Ø0.5-mm tube, which reach approximately 250 ms (Fig. 3G). Despite the variation, the FJ reaction is very fast, with most stiffness transitions occurring within 300 ms, which is at least two orders of magnitude faster than the reaction times of the existing VS catheters.

The robustness to leakage of the catheter is also investigated in view of safe operation inside the human body (see Materials and Methods). Underwater tests (fig. S11A) show that the catheters can undergo 160 cycles of stiffening and softening without leakage as no bubble is detected around the catheter and no water is detected inside the catheter tube. In addition, force tests also confirm no leakage as the catheter experiences no notable change in its stiffness in the soft and stiff states, with the contact forces remaining similar for the two states (fig. S11B). This means that our catheter, as a disposable device, is robust enough for the ablation surgeries, which usually require around 80 cycles of stiffness changes.

### Bending tests of the catheter under the RMN system

**Fig. 4.**
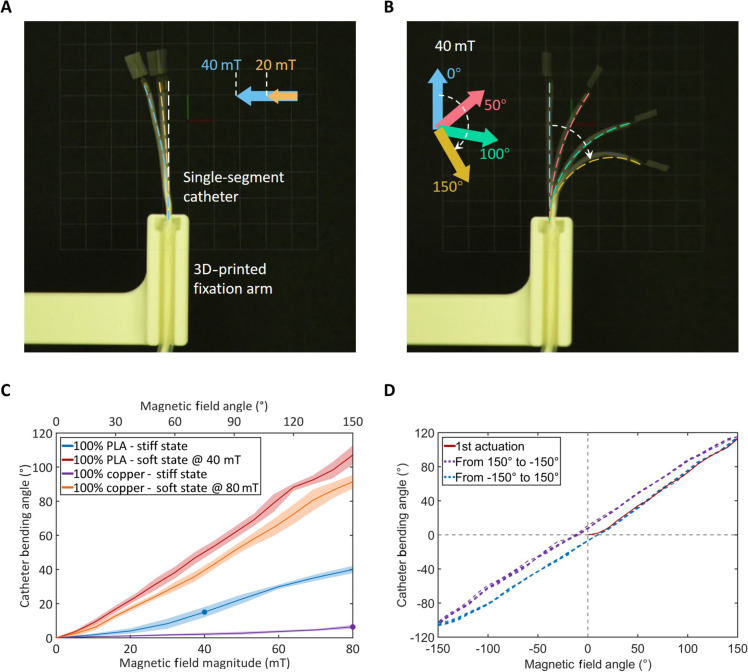
Single-segment catheter tests with the RMN system. (**A**) Catheter (100% PLA fibers of Ø50 μm, 45% filling rate) in the stiff state tested under increasing MFD with a fixed direction. The direction of the magnetic field is perpendicular to the initial orientation of the catheter body, generating maximum bending moments for the assessment of catheter rigidity. (**B**) Same catheter in the soft state tested under a fixed MFD with changing direction from 0 to 150°. (**C**) Bending results of the catheters with 100% PLA and 100% copper fibers from tests (A) and (B). The colored dots highlight the small deflections of the catheters in the stiff state using their corresponding MFDs. (**D**) Cyclic bending test result of the catheter with 100% PLA fibers to reveal its bending hysteresis.

After determining the optimal configuration for the FJ segment (45% filling rate, Ø50 μm) and the FJ reaction times, we performed bending characterization of the catheters with five different fiber compositions used in Fig. 3E with a hospital-compatible RMN system (CardioMag) to investigate how the catheters respond to the magnetic field in terms of bending angles in their soft and stiff states and the speed of shape locking. In the stiff state, the single-segment catheters are tested under an increasing magnetic field density (MFD) with a fixed direction perpendicular to the catheter to assess their rigidity (Fig. 4A). Subsequently, to investigate their bending range in their soft state, the catheters are tested under their corresponding MFDs, which is rotated from 0° (parallel to the catheter) to 150° (Fig. 4B). The MFDs for testing the catheters in their soft states are different due to different stiffness decided by their fiber material compositions, which are 40, 50, 60, 70, and 80 mT for 100% PLA, 75% PLA–25% copper, 50% PLA–50% copper, 25% PLA–75% copper, and 100% copper, respectively (section S4 and table S4).

In the stiff state, the catheters remain rigid at their recommended MFDs, with minimal deflections of 15.1°, 11.3°, 12.1°, 10.1°, and 6.4° at 40, 50, 60, 70, and 80 mT for the five fiber compositions, respectively (the highlighted dots in Fig. 4C and fig. S12, and movie S2). In the soft state, with the magnetic field turning 150°, the catheters with the five types of fiber bundles achieve large bending angles of 107°, 100°, 98°, 94°, and 92° for the five fiber compositions as compared to their deflections in the rigid state (Fig. 4C, fig. S12, and movie S2). In addition, a hysteresis of approximately 10° is observed in the cyclic bending tests (Fig. 4D and movie S2), which is primarily attributed to the fiber rearrangement during bending.

To demonstrate the instant stiffness change of the catheters, another cyclic bending is performed, in which the catheter is stiffened and softened multiple times (movie S3). We can see that the freezing and releasing actions of the catheter occur immediately upon turning the valve knob (movie S3). Overall, the calculation in section S4 gives accurate predictions of the MFDs for the single-segment catheters with different fiber compositions. The catheters under the RMN system, despite slight hysteresis, exhibit strong performance with small deflections in the stiff state, substantial bending in the soft states, and ultrahigh speed in stiffness change.

### Two-segment catheter for cardiac ablation

During open-volume surgeries, such as those involving the heart or stomach, the precise positioning of surgical tools requires two degrees of freedom. Thus, we develop a two-segment FJ catheter to address this need effectively. The first segment uses the 100% PLA fiber bundle with a low stiffness range to allow a wide bending range, while the second segment is filled with the 100% copper fiber bundles with a high stiffness range not only to allow adequate motion range but also to provide rigid anchoring for the first segment manipulation (section S4). Both segments use a 45% filling rate and Ø50-μm fibers. This two-segment catheter is then tested in the RMN system with the goals of demonstrating the selective manipulation of the two segments to achieve multi-curvature bending and examining whether the stiffened segment can remain unaffected when the other segment is being manipulated. Then, to simulate a real surgical scenario, the two-segment catheter is also tested in a 3D phantom of a human heart for manipulation by the RMN system.

**Fig. 5.**
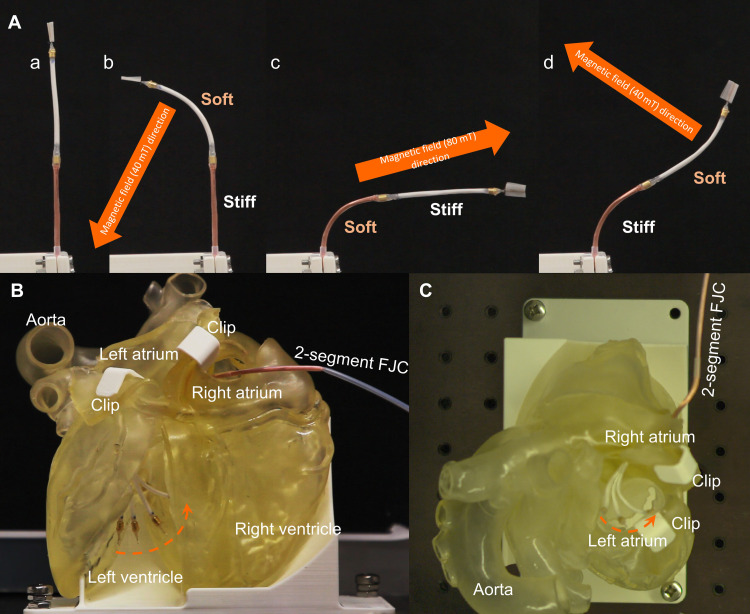
Two-segment catheter tests with the RMN system. (**A**) Selective manipulation of the two catheter segments to form a multi-curvature body. The initial state of the catheter is straight [(A), a]. The first segment is softened and manipulated, with the stiffened second segment staying still, in a 40-mT MFD magnetic field [(A), b]. The second segment is softened and manipulated, with the stiffened first segment staying straight, in an 80-mT MFD magnetic field [(A), c]. Following [(A), d], the second segment is stiffened, and the first segment is softened for manipulation to form a multi-curvature body in a 40-mT MFD magnetic field. (**B** and **C**) Catheter manipulation in a human heart phantom with the stiffened second segment supporting the manipulation of the first segment in the left ventricle (front view) (B) and left atrium (top view) (C). FJC, FJ catheter.

In the demonstration, the catheter is initially positioned vertically with the two segments stiffened (Fig. 5Aa). Then, the first segment is softened for manipulation by changing the direction of a 40-mT magnetic field. While the first segment is moved leftward, the second segment remains still, which validates the sufficient rigidity of the 100% copper fiber bundle in the vacuumed state (Fig. 5Ab and movie S4). In the second test, the second segment is manipulated to the right in an 80-mT magnetic field and the stiffened first segment stays straight during the procedure (Fig. 5Ac and movie S4). Although the rigid first segment cannot withstand the 80-mT MFD, the movement of the softened second segment occurs quickly before any deformation of the first segment occurs (movie S4). The second segment is then stiffened, followed by the softening of the first segment for upward manipulation with a 40-mT MFD, resulting in a multi-bending curve (Fig. 5Ad and movie S4). The multi-curving capability allows the catheter to substantially extend the workspace compared to the single-segment catheter (fig. S13). In addition to the multi-segment curving capability, the overall actions of the catheter are performed fluently with a whole set of curve forming, including three rounds of stiffness switching and individual bending of the two segments, taking less than 10 s. The other VS catheters using phase-changing materials require at least 10 min to accomplish the set of movements (*11*–*14*).

In the next demonstration, the two-segment catheter is inserted into a 3D phantom of the human heart via the right atrium, through the wall puncture between the left and right atriums, to the left ventricle. At first, we stiffen the second copper segment and manipulate the soft first segment using a 40-mT magnetic field for potential ablation procedure (Fig. 5B and movie S5). Next, the catheter is withdrawn to the left atrium and then, with the second segment frozen, the soft first segment is bent to different positions by the magnetic field (Fig. 5C and movie S5).

To demonstrate maneuverability, we steered the catheter through a 2D maze (fig. S14). By controlling the direction of the magnetic field, the catheter tip is pointed toward the chosen path and advanced toward the desired exit of the maze (movie S6).

The three demonstrative tests above validate the motion capability, effectiveness of the stiffness configuration in the two segments, and rapid VS function of the catheter, highlighting a promising potential for its use in cardiac ablation surgeries.

### Applied force characterization of a two-segment catheter

The quality of lesion formation during a cardiac ablation procedure is determined by a number of factors including the amount of power applied to the ablation tip, duration of ablation, and amount of force applied to the human tissue by the ablation tip (*28*). Currently, doctors apply at least 15*g* of force onto the human tissue with existing manually driven catheters for high-quality lesion formation (*29*). Existing clinical magnetically driven catheters can apply 6*g* of force only, which results in longer surgery duration and lower-quality lesion, leading to higher cases of cardiac arrhythmia recurrence (*30*–*32*). VS catheters have the potential to enhance the force applied to the human tissue, resulting in a high-quality lesion formation and higher success rate of the procedure. We characterized the applied force of our two-segment FJ catheter in clinical setting using a commercially available RMN system (Niobe, Stereotaxis) (fig. S15, A and B) and compared its performance with a commercial catheter (Celsius RMT Thermocool, Biosense Webster J&J). First, our catheters were extended from 50 cm using additional tubing to form a standard-length cardiac ablation catheter of 135 cm. The additional tubes, despite the extended length, were able to transmit the applied negative pressure from the vacuum pump to the VS segments. During force tests, the catheter was then installed onto a support setup. The extended tubes were inserted into an advancing setup, which enables the linear movement of the catheter. Simultaneously, the bending angle of the catheter was controlled using a magnetic field generated by the permanent magnets of the RMN system. The magnetic field was regulated by the experienced surgeon using the control panel of the robotic system. We used three force application scenarios commonly used by clinicians during ablation surgeries: perpendicular push onto the human tissue using only the catheter advancer, lateral touch with an object parallel to the catheter using only the magnetic torque, and lateral touch with an object parallel to the catheter using both catheter advancer and magnetic torque (see Materials and Methods).

Overall, our FJ catheter in stiff mode applies forces comparable to the commercial catheter, though with slightly larger SDs (Table 1). In the perpendicular push scenario (fig. S15, C and D), the stiffened FJ catheter can apply more than 8*g* of force, 41% larger than the commercial catheter. In the lateral touch scenario with only magnetic guidance (fig. S15E), the commercial catheter applies a force of 2.03*g* compared to 0.76*g* of the FJ catheter. This difference is mainly because our FJ catheter has only one magnet at the foremost tip, whereas the commercial catheter has four magnets. Consequently, we expect the performance of our catheter in stiff mode to be comparable or superior to the commercial catheter when equipped with four magnets at the tip. Finally, in the lateral touch scenario with both magnetic guidance and catheter advancer (fig. S15F), both catheters display comparable performance, although in this case too we expect the performance of our catheter to be superior if equipped with four magnets at the tip.

**Table 1.** Summary of applied force results.

Catheter	Test scenario					
	Perpendicular push (g)	SD (g)	Lateral touch (g)	SD (g)	Lateral touch and push (g)	SD (g)
Fiber jamming catheter (soft state)	4.02	0.215	0.43	0.207	2.32	0.093
Fiber jamming catheter (stiff state)	8.20	0.378	0.76	0.333	3.25	0.139
Celsius RMT Thermocool	5.85	0.063	2.03	0.045	3.34	0.167

In the soft state, our catheter yields half the force of the stiff state (4.02*g*), 31.3% lower than the commercial catheter, suggesting that it can be safer during navigation in the body than existing certified devices while applying comparable or higher force to certified catheters in stiff mode and thus offer high-quality lesion formation. Furthermore, the VS catheter, in both stiff (8.2 N) and soft (4.02 N) modes, does not exceed the perforation force threshold of 77*g*, offering the potential for safer and improved surgical outcomes (*29*).

### Ex vivo ablation demonstration

To demonstrate the ablation capabilities of our catheter in both soft and rigid states, we conducted an ex vivo test using animal tissue. A commercially available heating element was placed at the catheter tip and encapsulated with silicone glue (fig. S16A). Two electric wires, each with a diameter of 50 μm, were passed through the working channel and connected to the power supply. The catheter was mounted on the linear stage of the Instron machine to allow vertical mobilization toward the tissue. In both the rigid and soft states, the catheter tip was advanced 3 mm downward to press against the tissue. Afterward, a power of 5 W was applied to the 5-ohm ablation tip (fig. S16B and movie S7).

The lesions formed after 13-s ablation measured 12 mm × 6 mm in the rigid state and 8 mm × 4 mm in the soft state (fig. S16, C and D). The depth and size of lesion formation depend on time, power, and the force applied (*29*). Since the catheter in the rigid state is less prone to buckling, it conveys higher force that results in a larger lesion.

## DISCUSSION

VS is a highly sought-after feature in MISs, such as cardiac ablation, due to its potential to substantially enhance the dexterity of the device and increase applied force during ablation, ultimately improving the success rate of a surgery. Although numerous VS mechanisms exist, including mechanical locking devices (*33*), magnetic/electric fields, and electrostatic-based approaches (*34*, *35*), few are suitable for MIS applications due to constraints related to size, stiffness range, and safety.

Here, we proposed and characterized a multi-segment VS catheter based on FJ that can be used in RMN procedures. The proposed catheter can reversibly change stiffness up to 6.5 times in only 600 ms, which is 300 times faster than other VS catheters (*11*–*14*).

Although the SCF of this catheter is three times lower than that of existing VS catheters that use phase-changing materials, the experimental results described here indicate that its bending performance in rigid and soft states is comparable.

The proposed fabrication method can be adapted for manufacturing catheters of different lengths, thicknesses, stiffness, and segment numbers that are suitable for other types of medical procedures, such as monitoring and surgery of the abdominal or of the otorhinolaryngological pathways that require catheters of different diameters, lengths, and segment numbers (*36*, *37*).

VS allows FJ catheters to apply 4*g* and 8.2*g* of force in soft and stiff states, respectively. Compared to an existing commercial catheter, which applies 5.85*g* of force, our catheter holds the promise to be safer during insertion and navigation in soft state and more efficient during ablation due to comparable or higher forces in rigid state.

Cardiac ablation surgeries are intensive procedures that typically consist of dozens of ablation points at multiple sites (*38*). Although multi-segment VS catheters could facilitate the positioning of the ablation tip at the desired location, the transition time of existing VS catheters (*11*–*14*) requires approximately 1.5 min for one stiffness change cycle and 3 min for one curve formation, which requires the sequential stiffness change of each segment.

A typical surgery performed with conventional catheters of up to 80 ablations requires approximately 65 min (*38*). Existing VS catheters would increase this length by at least 240 min (90 s per stiffness transition cycle × 2 segments × 80 ablations). Instead, the catheter described here requires only a few minutes of additional operation time in today’s procedure, as the minimum time for our catheter to complete the same amount of manipulation is 1.6 min (600 ms per stiffness transition cycle × 2 segments × 80 ablations). Together, these results indicate that the proposed catheter not only offers performance comparable to other VS catheters and is compatible with the RMN system but also does not substantially increase operation time. Surgery duration is indeed a critical factor for the adoption of VS technologies in MISs and in other medical procedures.

Here, we described in vitro evaluation of the catheter’s dexterity and applied force using a static phantom of a human heart and magnetic field–compatible load cell, respectively. In future designs, the FJ catheter will feature the standard 135-cm length and incorporate additional magnets at the tip, increasing magnetic torque for greater applied force. Finite element simulations will also be investigated for better prediction of FJ performance. We will then assess the catheter performance in a more dynamic experimental setting with a beating heart phantom and circulating flow.

## MATERIALS AND METHODS

### Catheter fabrication

The first consideration in the fabrication is the ultrathin fiber. The only off-the-shelf fibers with different diameters are the enameled copper wires, which have an elastic modulus of 110 GPa. The other type of fiber is obtained by thinning the filament from a 3D printer (Ultimaker S5, Ultimaker B.V.) using winding machine I (Fig. 2A). Since the various filaments have similar elastic moduli of around 3 GPa, PLA is chosen due to its biocompatibility. During PLA fiber production, winding machine I pulls the melted PLA filament with its spinning spool. With a spinning speed of 12.5 πrad/s, the winding spool (Ø100 mm) can pull the melted PLA filament from the printer nozzle into Ø50-μm fibers, while other fiber thicknesses can be achieved via different spinning speeds (section S3 and table S5). The fiber production is fairly precise with around 5-μm SD in the fiber diameters. PLA fibers thinner than Ø50 μm can also be produced via the aforementioned process, but they are too brittle for later operation. This fiber production method with the use of 3D printer is potentially applicable to most 3D-printable thermoplastic materials that, when heated to their fluidic states by the printer, reach a viscosity similar to that of PLA at 200°C (around 10,000 Pa.s) (*39*). Although the fibers display a curvature after production due to the winding around the circular spool, they do not cause a bending of the catheter. This is because the fibers are not aligned when inserted into the silicone sleeve, and thus, the individual curvature of the fibers will cancel out, resulting in an overall straight catheter. To prepare fiber bundles with specific numbers of fibers, the winding frame from winding machine I is mounted on winding machine II, which rotates the winding frame at a slow speed (0.4 πrad/s) to avoid breaking the ultrathin fibers (Fig. 2B).

The silicone sleeve is prepared via a dipping-curing process (Fig. 2C). Multiple Ø2-mm steel rods fixed on the rod holder are slowly dipped into the freshly mixed silicone (DragonSkin 0020, Smooth-On Inc.) in the measuring cylinder for 5 s and then pulled out for 2-hour curing at room temperature on the curing stand. With the gravity effect, the dipping and curing process results in a consistent wall thickness (≈150 μm) on the silicone coating, which is a proper thickness for the sleeve. The silicone coating is rubbed off from the steel rod, and the upper 60-mm segment is kept as the sleeve for the FJ segment.

With all the components ready, the fabrication starts with the construction of the second FJ segment (fig. S4A). The silicone sleeve is first inserted into the right side of the PTFE tube, followed by the insertion of the assistive tube from the right into the silicone sleeve and then PTFE tube (fig. S4Aa). This assistive tube with a wedged tip is to facilitate the insertion of the fiber bundle due to its smooth inner surface. Then, the copper fiber bundle is inserted from the right, followed by the insertion of the working and vacuum channels (fig. S4Ab). The two thin channels, due to their softness, are internally reinforced by Ø0.3-mm assistive CF rods for easy insertion. The insertion of the two channels is done with the copper fibers held on the right end. Then, the copper fibers are released to fully enter the silicone sleeve along with the further insertion of the two channels (fig. S4Ac). With the removal of the assistive tube, silicone glue is then applied at the right tip of the silicone sleeve (fig. S4Ad), followed by the immediate insertion of the magnet and then the supporting CF rod (fig. S4Ae). Extra silicone glue is then applied to seal the second segment (fig. S4Af).

The next procedure is to produce the first FJ segment (fig. S4B). The assistive tube is first inserted into the silicone sleeve, followed by the insertion of the PLA fiber bundle (fig. S4Ba). The halfway first segment is inserted onto the tip of the second segment with the working channel passing through the first segment (fig. S4Bb). The silicone sleeve is then rubbed leftward to reach the tip of the second segment. After removing the assistive tube, the silicone sleeve is fixed to the tip of the second segment with silicone glue (fig. S4Bc). The right end of the PLA fibers is fixed with silicone glue (fig. S4Bd), followed by the immediate insertion of the magnet (fig. S4Be). The tip of the first segment is then sealed with extra silicone glue (fig. S4Bf). By removing the two assistive CF rods in the working and vacuum channels, the catheter fabrication is completed.

The other end of the catheter, which is the PTFE tube with the working and vacuum channels inside, is connected to the rear tubing assembly (fig. S5). For making the rear tubing assembly, the silicone tube, polyurethane (PU) tube, barb fitting, and the cross fitting are first assembled (fig. S5A). Then, the working and vacuum channels inside the PTFE tube are inserted through the silicone tube, the barb fitting, and the PU tube. When they reach the cross fitting, the vacuum channel goes straight across the cross fitting, while the working channel is diverted to the fitting port pointing downward (fig. S5B), followed by the insertion of the PTFE tube into the silicone tube. Another three PU tubes are then plugged into three fitting ports, and the assembly is finished with silicone glue sealing the tips of outlets 1 and 2 (fig. S5C). Figure S5D illustrates the three ways (working, vacuum channels, and the PTFE tube) with a cross-sectional view, which better explain the working principle of the catheter. The working channel can be used for inserting multiple thin electric wires required for powering the ablation tip (fig. S17).

The fabrication of catheters with hybrid fibers follows the aforementioned steps too. The production of the hybrid fiber bundle starts with the winding of PLA fibers followed by the copper fibers. They are not evenly distributed (fig. S10), as there is no evidence that even distribution can improve the performance of the catheters.

### Catheter stiffness measurement

For the stiffness measurement, the single-segment catheter is placed on a 3D-printed three-point bending setup with two supporting bars at two ends (gap: 35 mm) and one indenter in the middle (fig. S7A). The indenter is connected to the loadcell of the Instron machine (Instron5965, Instron Inc.), which moves the indenter to press on the catheter. The catheter is connected to one channel of the dual-channel vacuum controller (fig. S7A). This vacuum controller, with two individual channels, can switch the vacuum channels on and off with two switch valves (VHK3, SMC Ltd.), regulate the vacuum levels with two vacuum regulators (IRV20, SMC Ltd.), and display the vacuum pressures with two vacuum indicators (ZSE20A, SMC Ltd.) (fig. S7A). Because of the cross-sectional deformation upon vacuum application (fig. S8), the catheters are tested at four orientations with 45° intervals. This is achieved via a 3D-printed fixation setup including the catheter fixation and fixation housing that is placed next to the three-point bending setup (fig. S7B). The catheter fixation has an octagonal body with four orientation indicators to allow the testing of different catheters at four orientations (fig. S7B). During the test, the catheter is pressed by the by 3-mm indenter at a compression rate of 0.2 mm/s. The compression distance, which is also the deflection of the catheter, and force data are recorded by the Instron machine at a 10-Hz sampling rate (fig. S7C).

For each catheter configuration with specific filling rates, fiber diameters, and fiber materials, three samples are fabricated. For each testing configuration with specific vacuum levels and fixed orientation, the tests are repeated three times for each catheter sample. After the tests, the force-deflection data within 1-mm deflection is used to calculate the stiffness via linear fitting. By dividing the stiffness in the stiff state by the stiffness in the soft state, the SCF is then calculated.

### Catheter reaction time measurement

The catheter reaction time measurement is also implemented on the three-point bending setup (fig. S7). During the test, the catheter is first compressed by the indenter by 3 mm (fig. S7C) and then, in this bent state of the catheter, the vacuum is turned on and off for five cycles. The force profile of the catheter transition between the soft and stiff states is obtained from the loadcell recording of the Instron machine (Fig. 3F). The force profile shows that the soft state gives higher force, while the stiff state yields lower force (Fig. 3F). This is because the vacuum inside the catheter shrinks the catheter body, which decreases its pressure to the indenter/loadcell. The vacuum also freezes the catheter body, which ensures that the catheter body does not lose its bent shape and thus maintains the low pressure on the loadcell. Therefore, the gaps between the last peak in the soft state and the first trough in the stiff state, and vice versa, represent the stiffening and softening times, respectively (Fig. 3F). The last three cycles of the force-time data are used to measure the reaction times. During the tests, the sampling rate is set at 1000 Hz to ensure precise time measurement.

For the reaction time measurement, 2-m-long tubes with different IDs (2 and 0.5 mm) are used to connect the catheters and the vacuum controller as, in the actual surgeries, the catheter should be long enough to extend from the vacuum controller to the ablation sites in the human heart, while 2- and 0.5-mm IDs are the ones used in the two-segment catheter to apply vacuum to the two segments. When using the 2-mm ID tube, the reaction times are measured from catheter samples with different filling rates, fiber diameters, and fiber materials. When using the 0.5-mm ID tube, only the three catheters with Ø50-μm PLA fibers at 45% filling rate are tested. The peak-to-trough interval for stiffening time and the trough-to-peak interval for softening time were chosen as the reaction time (Fig. 3F) instead of the peak-to-peak interval (see section S6 for the explanation).

### Catheter robustness assessment

The robustness test of the catheter is performed underwater using the three-point bending setup (fig. S11A). During the test, the catheter is first compressed by the indenter by 3 mm and then the vacuum is turned on and off for 160 cycles. The force profile of the catheter transition between the soft and stiff states is recorded by the loadcell of the Instron machine at 1000 Hz. Similar to reaction time measurements, the soft state of the catheter gives higher forces, while the stiff state yields lower forces (fig. S11B). The average forces from the soft and stiff states are then calculated for each cycle.

### Catheter bending test under RMN system

The bending tests of the single-segment catheters are implemented with the CardioMag RMN system. Five types of fiber bundles (100% PLA, 75% PLA–25% copper, 50% PLA–50% copper, 25% PLA–75% copper, and 100% copper) are used in the catheters with three samples for each fiber bundle type. All catheters are constructed with the optimal FJ configuration: 45% filling rate and 50-μm fiber diameter. Before the start of the tests, the catheter is adjusted into a straight body with the rear tube fixed on a 3D-printed fixation arm (Fig. 4, A and B). In the rigid/vacuumed state, the catheters are tested with the magnetic field directed perpendicular to the initial catheter body, representing the maximum bending moment that can be generated by each MFD. With camera recording, the MFD is increased from 0 to 80 mT with an incremental rate of 10 mT/3 s to bend the catheters. In the soft state, the catheters are tested with specific MFDs corresponding to different fiber bundle types: 40, 50, 60, 70, and 80 mT for 100% PLA, 75% PLA–25% copper, 50% PLA–50% copper, 25% PLA–75% copper, and 100% copper, respectively. During the tests, the magnetic field is initially aligned to be parallel to the catheter body, representing a 0 bending moment. The magnetic field is then rotated by 10° every 3 s until reaching an angle of 150° from its initial angle. When the magnetic field is rotating, the magnetic field direction misaligns with the catheter magnet, resulting in a bending moment to bend the catheter. Therefore, the bending of the catheter follows the rotation of the magnetic field. The bending processes are also recorded by a camera. For the cyclic bending tests to access the hysteresis, the catheters are tested in the soft state with their MFDs. With the camera recording, the magnetic field is rotated from its initial direction parallel to the initial catheter orientation, and then oscillates five times between −150° and 150° with a rotation rate of 10°/3 s. For each catheter sample, all the tests are repeated three times each. All the recorded videos are then processed by motion analysis software (Tracker, physlets.org) to measure the bending angles.

### Catheter force measurement

To measure applied force, we used a custom-made load cell, which allows force measurement inside the magnetic field with a precision of 0.01*g* (fig. S15, A and B). For the force tests, our catheters were extended from 50 cm to the full length of a standard catheter (135 cm) using additional tubing. To control the orientation, every catheter was fixed to a custom-made holding setup placed inside the working area of Niobe (fig. S15B). The setup connects a catheter advancer, support shaft, and the catheter. A catheter advancer (Quick-CAS, Stx) moves a catheter forward and backward inside the working area. The catheter advancer is connected to a steerable introducer (Agilis, NxT), which is used as a support sheath to deliver the catheter to a target zone inside the heart at a desired inclination angle. It also provides additional rigidity to the system since it has a larger diameter and higher stiffness compared to a catheter. By extending or retracting the catheter from the support sheath, doctors can tune the force applied to the human tissue during ablation (*32*). The catheter can be bent in any direction by the magnetic field (amplitude of 80 or 100 mT) generated by permanent magnets located at the left and right sides of the patient. The FJ catheter was tested in soft and stiff states (both segments soft or stiff) along with a Celsius catheter.

We performed characterization for three force application scenarios commonly used by clinicians at ablation surgeries: perpendicular push onto the human tissue using a catheter advancer only (fig. S15C), a lateral touch with an object parallel to the catheter using a magnetic torque generated by permanent magnets at the tip of the catheter and Niobe’s magnetic field (fig. S15E), and a lateral touch with an object parallel to the catheter using both a magnetic torque and forward motion of catheter advancer (fig. S15F). During all experiments, the catheter tip was pressed onto the load cell until it was bent and the force was constant (fig. S15D). The bent configuration of the catheter mimics a real case contact during the procedure but it does not represent the optimized configuration to apply maximum force with the device. We performed 20 measurements for each scenario and device (Table 1).

## Supplementary Materials

### The PDF file includes:

Sections S1 to S6 Figs. S1 to S19 Tables S1 to S6 Legends for movies S1 to S7
