# Supplementary material for: Instant variable stiffness in cardiovascular catheters based on fiber jamming
Source: Sci Adv. 2025 Feb 7;11(6):eadn1207. doi: 10.1126/sciadv.adn1207 (PMC11804929; doi:10.1126/sciadv.adn1207)
Supplement: Supplementary file 1 — Sections S1 to S6 Figs. S1 to S19 Tables S1 to S6 Legends for movies S1 to S7 [file sciadv.adn1207_sm.pdf]

Science Advances
AAAS

Supplementary Materials for

Instant variable stiffness in cardiovascular catheters based on fiber jamming

Yi Sun *et al.*

Corresponding author: Yi Sun, yi.sun@epfl.ch

Sci. Adv. **11**, eadn1207 (2025)
DOI: 10.1126/sciadv.adn1207

The PDF file includes:

Sections S1 to S6
Figs. S1 to S19
Tables S1 to S6
Legends for movies S1 to S7

Other Supplementary Material for this manuscript includes the following:

Movies S1 to S7

Section S1. SCF requirement for VS catheter

SCF is used to define the VS performance, which is given as

$$SCF = \frac{K_r}{K_s} \quad \text{(S1)}$$

where K_r and K_s are the stiffness of the VS catheter in the stiff/rigid and soft states, respectively.

The stiffness of the catheter is obtained using the 3-point bending test (fig. S18A).

$$K = \frac{F}{y} = \frac{48EI}{L^3} \quad \text{(S2)}$$

where F is the force applied to the catheter. L is the distance between the two lateral supports of the 3-point bending test setup. y, E and I are the middle deflection, elastic modulus and second moment of area of the catheter, respectively.

Therefore,

$$SCF = \frac{K_r}{K_s} = \frac{\frac{48EI_r}{L^3}}{\frac{48EI_s}{L^3}} = \frac{I_r}{I_s} \quad \text{(S3)}$$

where I_r and I_s are the second moment of area of the catheter in the rigid and soft states, respectively.

When the catheter is placed in the magnetic field, the bending moment of the magnetic field M will cause deflection δ in the catheter (fig. S18B).

$$\delta = \frac{Ml^2}{2EI} \quad \text{(S4)}$$

where l is the length of the catheter.

Therefore,

$$SCF = \frac{I_r}{I_s} = \frac{\frac{Ml^2}{2E\delta_r}}{\frac{Ml^2}{2E\delta_s}} = \frac{\delta_s}{\delta_r} \quad \text{(S5)}$$

where δ_r and δ_s are the deflections of the catheter in the rigid and soft states, respectively.

The general requirement for the VS catheter is that, under the same magnetic field, its deflection in the rigid state δ_r should be less than 25% of the deflection in the soft state δ_s. Therefore, the desirable SCF the VS catheter should achieve is 4 and above.

$$SCF = \frac{\delta_s}{\delta_r} \geq 4 \quad \text{(S6)}$$

Section S2. Simplified FJ modeling of the relation between SCF, fiber size and fiber number.

When bending the catheter in the stiff and soft states with the same method, for example, the 3-point bending method (fig. S18A), the SCF will be the ratio of the second moments of area of the catheter in the stiff and soft states as given in equation S3.

For the FJ catheter, we can simplify it as a fiber bundle with N fibers confined in a cylindrical space with a circular cross-section area at A. In the stiff state, we assume there is no relative sliding between the fibers, and therefore the fiber bundle can be considered a solid beam. Therefore,

$$I_r = \frac{\pi R^4}{4} \quad \text{(S7)}$$

where R is the radius of the cross-section circle of the FJ beam, which is $\sqrt{\frac{A}{\pi}}$.

In the soft state, we assume that the fibers in the fiber bundle can bend freely around their own central axes. Therefore,

$$I_s = N \cdot \frac{\pi r^4}{4} \quad \text{(S8)}$$

where N is the number of fibers and r is the radius of the single fiber, which can be estimated as $\sqrt{\frac{A}{N\pi}}$.

Therefore,

$$SCF = \frac{I_r}{I_s} = \frac{\frac{\pi R^4}{4}}{N \cdot \frac{\pi r^4}{4}} = \frac{\frac{\pi \sqrt{\frac{A}{\pi}}^4}{4}}{N \cdot \frac{\pi \sqrt{\frac{A}{N\pi}}^4}{4}} = N \quad \text{(S9)}$$

Therefore, a higher SCF of the FJ beam can be achieved with a larger number of fibers with thinner thickness.

Section S3. PLA fiber production

PLA fibers are produced by winding machine I pulling melted PLA filament from the nozzle of an Ultimaker 3D printer. The printer does not have the function to extrude PLA, however, during the material loading process, the printer will extrude melted filaments at a fixed speed for up to 3 minutes, which allows us to obtain enough amount of fibers. When the printer head starts to extrude the PLA filaments, the initially extruded filament will be quickly attached to the spool of winding machine I. Then winding machine I is started and the spool starts spinning from 0rad/s to the set velocity with a 5s linear acceleration. Without this gentle acceleration, the high speed of the spinner will immediately break the soft filament.

Due to the high speed of the spool and the slow extrusion of the melted PLA filament, the spool will pull the initially thick strand into very thin fibers. By changing the spinning speed of the spinning spool (Ø100mm), PLA fibers of different diameters can be obtained. Through testing different spool spinning speeds and measuring the resultant fiber diameters (Table S5), we obtained fibers with diameters of 50µm, 75µm and 50µm for making the catheters that are used in the characterization.

Section S4. Stiffness configuration of the FJ segments of the multi-segment catheter.

The FJ VS catheter will be equipped with two segments to achieve multi-curvature bending to reach more challenging surgical sites. When the stiffened catheter with two segments made of the same fiber configuration is placed in the magnetic field, the first segment is subject to one bending moment M, while the second segment is subject to a tripled moment $3M$ (fig. S18C). Due to the relatively low stiffness of FJ segment, when the first segment is just stiff enough to withstand the bending moment, the second segment will not be able to withstand the tripled moment. Therefore, the two segments cannot have the same stiffness range and it is necessary to customize the stiffness ranges of FJ segments. A viable choice is to assign a low stiffness range to the first segment and endow the second segment with a high stiffness range.

Hybrid fiber bundles by mixing fibers of two different materials and stiffness with different ratios provide a solution for stiffness customization, and the experimental results show that the stiffness ranges of the fiber bundles change with different PLA and copper fiber percentages (Fig. 3E). The characterization results from the pure PLA and copper bundle along with the three hybrid fiber bundles will be used to evaluate their potential performance in the RMN system and then to design the two FJ segments in the two-segment catheter.

To build the connection between 3-point bending and the RMN performance of the FJ catheter, we can use the flexural rigidity EI of the catheter as both performances rely on the same EI. The deflection-force relation of the 3-point bending is given as

$$y = \frac{FL^3}{48EI} \quad \text{(S10)}$$

where *y*, *F* and *L* are the middle deflection of the catheter, the force applied by the middle indenter, and the distance between the two lateral supporting points in the 3-point bending tests, respectively (fig. S18A); *E* and *I* are the overall elastic modulus and second moment of area of the catheter, respectively. *EI* makes the flexural rigidity.

Therefore, with the experimental results from the 3-point bending tests, we can calculate *EI* using the equation below:

$$EI = \frac{FL^3}{48y} \quad \text{(S11)}$$

The deflection of the catheter in the magnetic field is given as

$$\delta = \frac{Ml^2}{2EI} \quad \text{(S12)}$$

where δ is the deflection at the tip of the catheter; *M* is the torque generated by the magnetic field that is applied to the magnet; *l* is the length of the catheter; and *EI* is the flexural rigidity that has been obtained from the 3-point bending.

The magnetic torque can be calculated as

$$M = m \times B \quad \text{(S13)}$$

where *m* is the dipole moment of the permanent magnet mounted at the tip of the catheter, which is 0.01187Am^2; *B* is the MFD generated by the RMN system, which ranges from 0 to 80mT.

Therefore we can calculate the MFD needed to achieve a given deflection.

$$B = \frac{2EI\delta}{ml^2} \quad \text{(S14)}$$

The experimental results of the 3-point bending of the five types of fiber bundles and their estimated performance in the RMN system are given in Table S4. For the soft and stiff states of the five types of fiber bundles, we calculate the MFDs needed to achieve small deflection (5mm) and large deflection (15 and 20mm). Please note that the equations are only valid for small deflections and the MFD calculations for two large deflections (15 and 20mm) are given as a rough reference.

To analyze the results, we take the 100% PLA fiber bundle as an example. Ideally, the FJ catheter needs to be bent with large deflections in the soft state under the magnetic field and stay within small deflections in the stiff state. From Table S4, we can see that the soft 100% PLA bundle needs an MFD of around 26 to 35mT to achieve large deflections, while its stiff state requires 58mT to reach 5mm deflection, which is larger than 35mT. Therefore, the 100% PLA fiber bundle is eligible for the catheter application, and so are the other four types of fiber bundles as their MFDs needed for small deflection in their stiff states are higher than the MFDs for large deflections in their soft states. With the calculation, the recommended MFDs for each fiber bundle type in the soft state are given as 30, 50, 60, 70 and 80mT for 100% PLA, 75% PLA – 25% copper, 50% PLA – 50% copper, 25% PLA – 75% copper and 100% copper, respectively.

The next is to consider the material configuration for the two-segment catheter. As suggested at the beginning of section S4, the first segment should have a low stiffness range, therefore, the 100% PLA fiber bundle can be assigned to the first segment. The second segment, when stiffened, needs to be rigid enough to support the manipulation of the first segment with minimal deflection. In this scenario, the second segment is subject to a tripled moment which is equal to the condition when placed in the magnetic field with a tripled MFD (fig. S18C). Therefore, the stiff second segment needs to withstand an MFD three times stronger than that needed for the soft 100% PLA bundle to achieve a large deflection, which is around 90mT. Therefore, only 25% PLA – 75% copper and 100% copper fiber bundles are qualified for the second segment. For guaranteed performance, the 100% copper fiber bundle is used in the second segment of our two-segment catheter.

As mentioned above, the 100% PLA fiber bundle is chosen for the first segment of the two-segment catheter. In the surgical scenario, the first segment will be in direct contact with the surgical points and thus prioritize larger bending angles for more working space and higher dexterity. Therefore, although the recommended MFD is 30mT, 40mT is chosen for the testing of the single-segment catheter with 100% PLA fiber bundle in the RMN system as 40mT will yield a larger deflection in the soft state of the catheter despite a slight compromise in the deflection in the stiff state.

Section S5. Simplified stiffness model.

We developed a simplified model based on the following assumptions: 1) In the soft state, there is no friction between the fibers, the silicone sleeve and the two internal tubes (the working and vacuum channels); 2) In the stiff state, the fiber bundle is reduced to a solid thick single-strand fiber with a cross-section area equal to the sum of the cross-section area of all the individual fibers (fig. S18D); 3) In both states, the silicone sleeve and internal tubes remain tubular.

Assuming a 3-point bending system, the stiffness of the fiber jamming segment is given in equation S2. Therefore, the stiffness of the catheter with different components can be given as follows:

$$K = \frac{48(EI_{fiber}+EI_{silicone}+EI_{tubes})}{L^3} \qquad \text{(S15)}$$

where EI stands for flexural rigidity, the subscripts *fiber*, *silicone* and *tubes* indicate PLA fiber, silicone sleeve and internal tubes, and L stands for the distance between the two lateral supports of the 3-point bending test setup.

For 45% of filling rate with Φ50μm PLA fibers, there are 720 fibers in the soft state and the flexural rigidity of each fiber is 8.48×10^{-10} Pa·m^4, therefore, $EI_{fiber} = 720\times8.48\times10^{-10} = 6.1\times10^{-7}$ Pa·m^4; $EI_{silicone} = 2\times10^{-7}$ Pa·m^4; $EI_{tube} = 2\times1.22\times10^{-6} = 2.44\times10^{-6}$ Pa·m^4. The overall flexural rigidity in the soft state, $EI_{soft} = EI_{fiber}+EI_{silicone}+EI_{tube} = 3.25\times10^{-6}$ Pa·m^4. Therefore, with 35mm L, the stiffness of the catheter in the soft state $K_{\text{soft}} = 48\cdot EI_{soft}/L^3 = 3.64$ N/m = 3.64 mN/mm;

In the stiff state, the diameter of the single thick fiber strand $D_{fiber*} = 2\cdot\sqrt{720\cdot\left(\frac{0.05}{2}\right)^2}$=1.3mm; and $EI_{fiber*} = 4.4\times10^{-4}$ Pa·m^4; and overall flexural rigidity in the stiff state, $EI_{stiff} = EI_{fiber*} + EI_{silicone} + EI_{tube} = 4.42\times10^{-4}$ Pa·m^4; and thus the stiffness in the stiff state $K_{\text{stiff}} = 48\cdot EI_{stiff}/L^3 = 494.9$ mN/mm. The calculated and measured results are given in Table S6.

From Table S6, we can see that the measured stiffness is located inside the theoretical range, meaning that the experimental results are reasonable. However, there are clear discrepancies between the measurement and model values. In the soft state, the experimental stiffness is five times greater than the value obtained from the model. In our opinion, this discrepancy can be explained from our assumption that the friction between fibers is zero in the soft state. However, in reality, the friction in the soft state may depend on several factors, such as the filling rate, fiber thickness, fiber roughness, precise geometry of the fibers and their alignment relative to each other. For example, higher filling rates are expected to increase friction between fibers; and, given a filling rate, thinner fibers would correspond to a higher number of fibers thus leading to a larger number of contacts and higher friction. Also, due to the randomness of the fiber arrangement, there could be different combinations of point and line contacts among fibers, which affect the stiffness too. An alternative method for predicting the FJ performance could be the use of finite element simulations.

In the stiff state, the experimental stiffness obtained within 0.2mm deflection which represents the stiffness without fiber sliding is less than half the calculated stiffness. The lower experimental value is mainly caused by the random arrangement of fiber which might loosen the fiber contact.

Therefore, our understanding is that the fiber jamming segment is a highly coupled system as a function of fiber number, fiber thickness, filling rate, fiber material, surface roughness of the fiber and other uncertainties. Its complexity and underlying theories may require the length of a new article for thorough explanation and validation.

Regarding the silicone sleeve in the model, please note that, in the stiffened state, the flexural rigidity of the silicone sleeve ($EI_{silicone} = 2\times10^{-7}$ Pa·m^4) is three orders of magnitude smaller than that of the stiffened fiber ($EI_{fiber*} = 4.4\times10^{-4}$ Pa·m^4) and thus may be considered negligible. However, in the soft state, the flexural rigidity of the silicone sleeve is of a similar magnitude to that of the fiber ($EI_{fiber} = 6.1\times10^{-7}$ Pa·m^4) and the thin PTFE tubes ($EI_{tube} = 2.44\times10^{-6}$ Pa·m^4), indicating that it significantly affects the stiffness of the catheter in the soft state. Since the silicone sleeve is a constituent element of the catheter, it is important to include it even if it complicates the model.

Section S6. Reaction time measurement method.

Reaction time should be measured as peak-to-peak intervals (Fig. 3F). However, such measuring method can have large errors. Using the stiffening process as an example, the force measurements often display large time gaps between the first trough and the first peak at the beginning of the low force level (fig. S19). These time gaps were mainly caused by the measurement noise of the instrument that occurred in a random manner at different wavelengths. In the specific case shown in fig. S19, the correct reaction time should be around 120 ms (from black to yellow dashed line). Our method results in 230 ms (from black to green dashed line) which has already included a substantial amount of error. If we use peak-to-peak intervals, the reaction time is around 375 ms (from black to pink dashed line) which has an even larger error. We also tried to filter the force curves, but it would flatten the peaks and troughs, making it hard to decide the start and end points of the measurement. Therefore, we chose to use the peak-to-trough (stiffening) and trough-to-peak (softening) intervals from the raw force data as the reaction time.

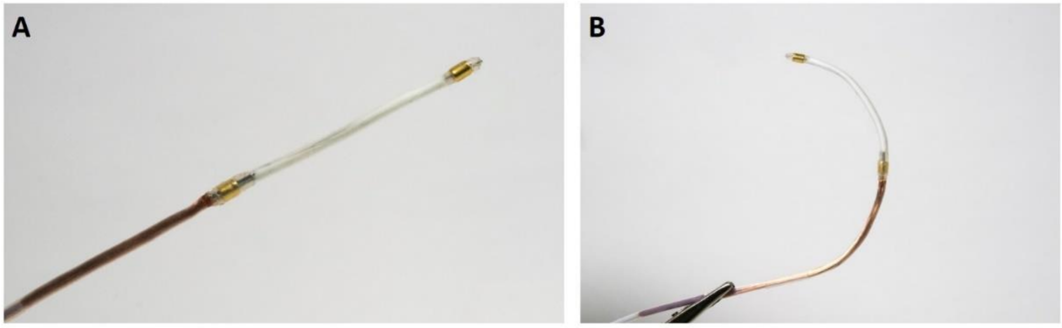

Fig. S1. Close-up views of the two-segment fiber jamming catheter. (**A**) Straight. (**B**) Curved.

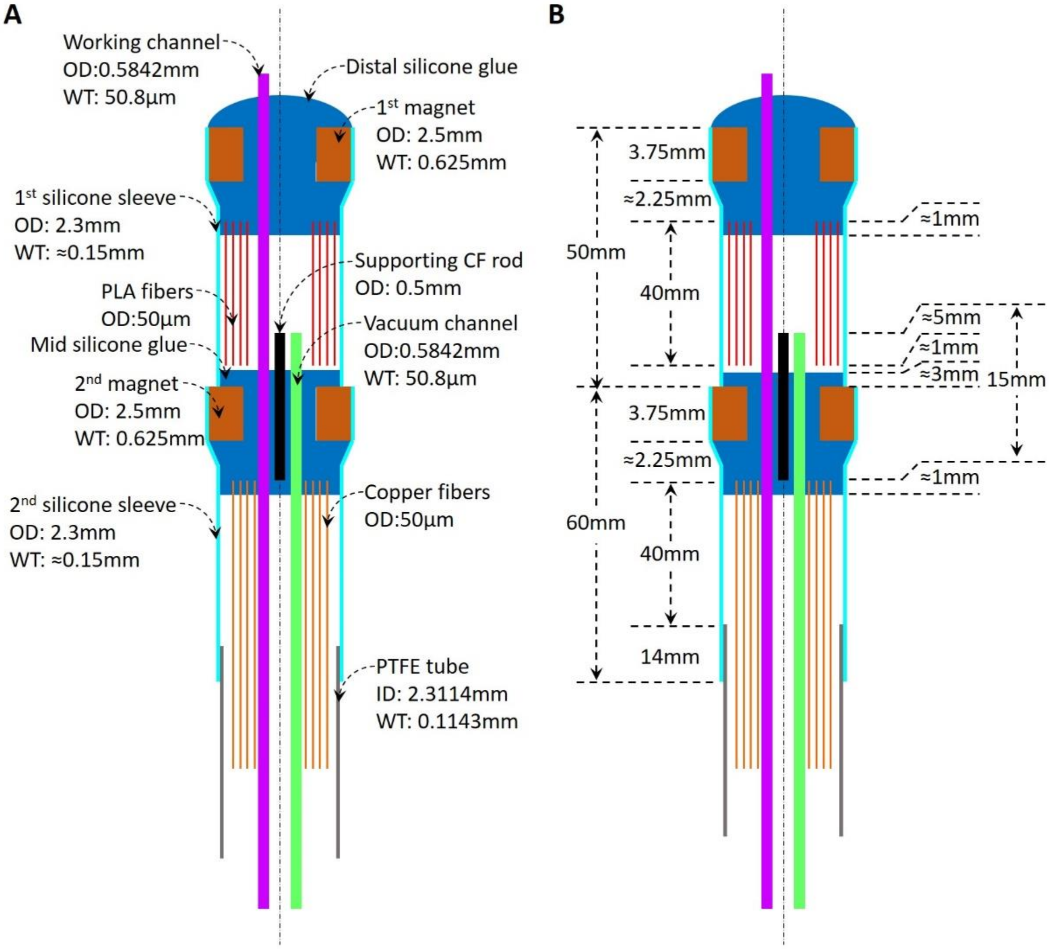

Fig. S2. The structure of the two-segment FJ VS catheter. (**A**) Illustration of components with diameters and wall thicknesses. (**B**) Component lengths and their relative positions.

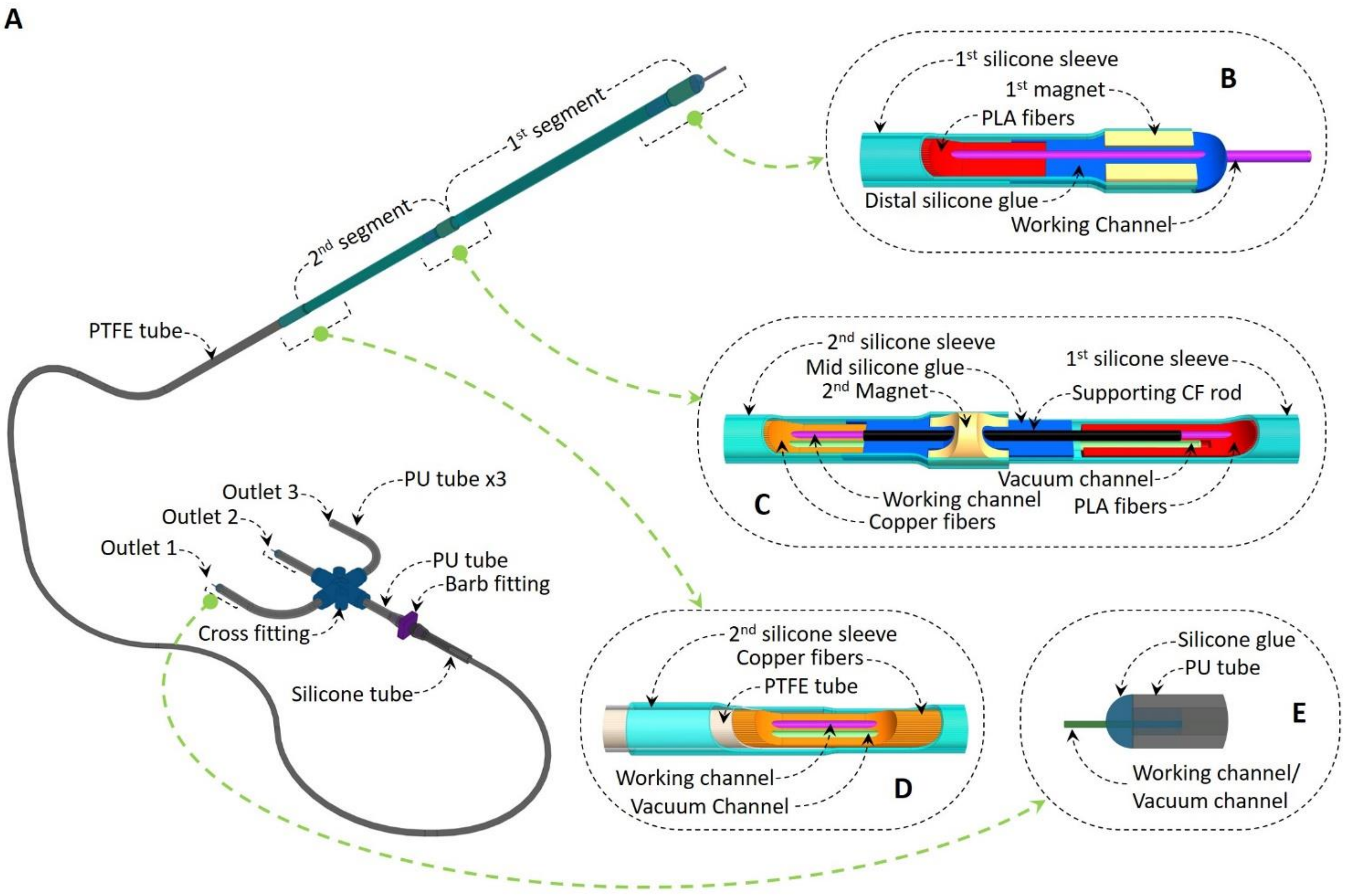

Fig. S3. Anatomy of the two-segment FJ VS catheter with true proportions. (**A**) Overview of the catheter with details of the rear tubing assembly. Near the rear tubing assembly, the PTFE tube is friction-fitted to a short silicone tube that is plugged into the 3.9-mm end of the barb fitting. The other end of the barb fitting (2.4mm) is connected to a short PU tube (ID: 2mm; OD: 4mm), which then goes to a cross fitting for tubing split into outlet 1, 2 and 3 for the placement of electric wire of the ablation tip, and vacuum application for the first and second FJ segments, respectively. (**B**) Dissection view of the tip of the first segment. (**C**) Dissection view of the middle connection between the two FJ segments. (**D**) Dissection view of the rear connection between the second segment and the PTFE tube. (**E**) Close-up view of the outlet showing that, to fully isolate outlet 1 and 2 from outlet 3, silicone glue is applied to block the PU tubes.

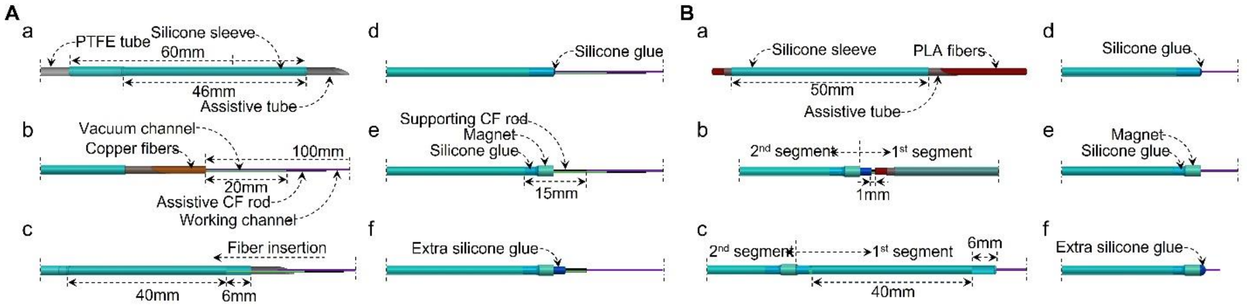

Fig. S4. Fabrication of the two-segment catheter with true proportions and key dimensions. (**A**) Second segment fabrication. The PTFE tube is inserted into the 60mm long silicone sleeve by 14mm, leaving 46mm of the silicone sleeve for the second segment. The assistive tube with a wedged tip is then inserted (a). Copper fibers are inserted from the right, followed by the insertion of the working and vacuum channels with 100mm of the working channel and 20mm of the vacuum channel sticking out of the copper fibers (b). Copper fibers are further pushed in with a 40mm length staying out of the PTFE tube and 6mm empty space at the tip of the silicone sleeve (c). The assistive tube is then removed, followed by the silicone glue application at the tip of the silicone sleeve (d). The magnet and the supporting CF rod (15mm long) are immediately inserted after glue application (e). Extra silicone glue is applied to seal the second segment (f). (**B**) First segment fabrication. The assistive tube with a wedged tip is inserted into the 50mm long silicone sleeve, followed by the insertion of the PLA fibers (a). The halfway first segment is inserted onto the tip of the second segment with a gap of around 1mm (b). The silicone sleeve of the first segment is rubbed onto the tip of the second segment, followed by the removal of the assistive tube and trimming of the PLA fibers down to 40mm. The silicone sleeve is then fixed using a small amount of silicone glue to the tip of the second segment (c). The tip of the first segment is filled with silicone glue to fix the PLA fibers (d). The magnet is inserted immediately after the silicone glue application (e). Extra silicone glue is applied at the tip to seal the first segment, followed by the removal of the assistive CF rods in the working and vacuum channels (f).

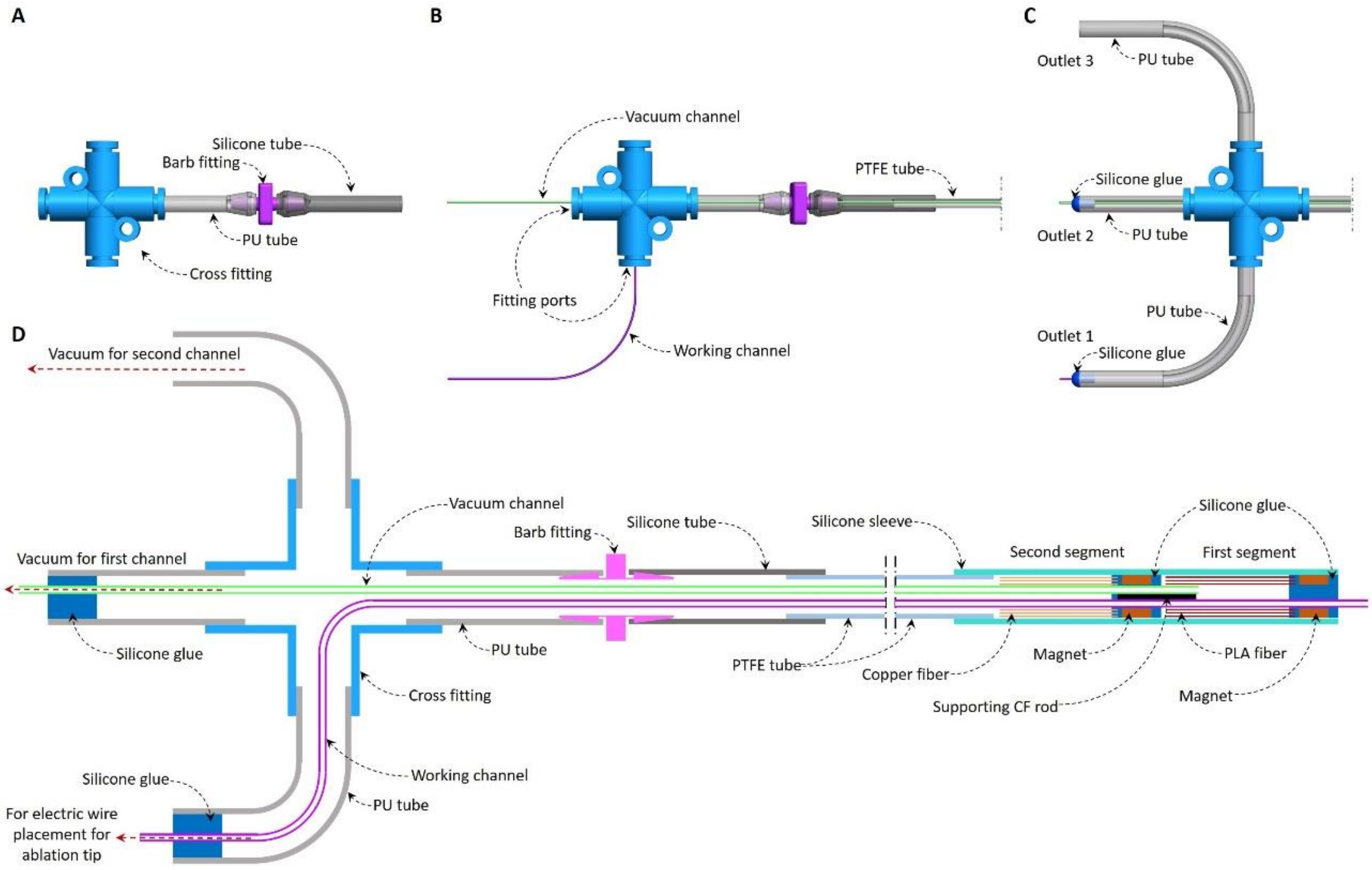

Fig. S5. Rear tubing assembly. (**A**) The silicone tube, PU tube, barb fitting and the cross fitting are first assembled. (**B**) The working and vacuum channels inside the PTFE tube are inserted through the silicone tube and, when they reach the cross fitting, the vacuum channel goes straight across the cross fitting, while the working channel is diverted to the fitting port pointing downwards, followed by the PTFE tube insertion into the silicone tube. (**C**) Three additional PU tubes are then plugged into three fitting ports and the assembly is finished with silicone glue sealing of outlet 1 and 2 tips. (**D**) This cross-section view of the rear tubing assembly illustrates the tube connection, airways for vacuum application in the first and second segments, and the working channel for potential incorporation of an ablation device.

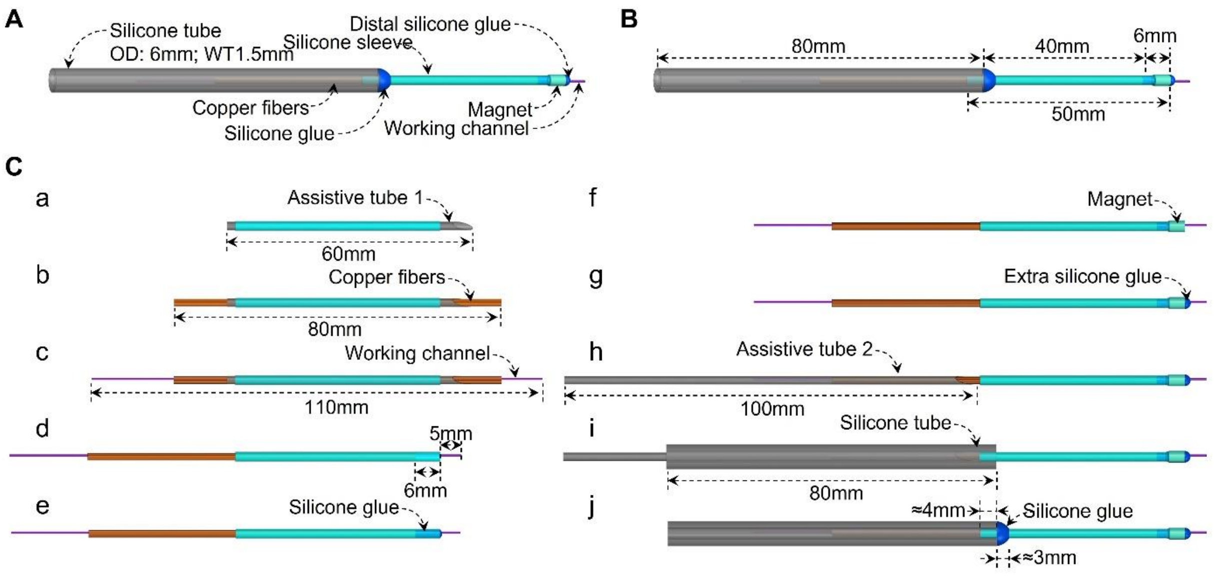

Fig. S6. Fabrication of the single-segment catheter. (**A**) The components used in the single-segment catheter. (**B**) Key dimensions for the single-segment catheter. (**C**) The fabrication processes of the single-segment catheter with copper fibers. The assistive tube 1 with a wedged tip is inserted into the silicone sleeve (a), followed by the insertion of the copper fibers (b). The working channel is then inserted (c), and then the fibers and the working channel are repositioned to leave 6mm empty space at the tip of the silicone sleeve and 5mm of the working channel sticking out of the silicone sleeve (d). Silicone glue is then applied to the tip of the silicone sleeve (e), followed by the immediate insertion of the magnet (f). Extra glue is then applied to seal the tip (g). The excessive copper fibers on the other end are inserted into the assistive tube 2 with a wedged tip (h), which is then inserted into a silicone tube with 4mm of the silicone sleeve inside the silicone tube (i). With the removal of the assistive tube 2 and the silicone glue application to fix the silicone sleeve with the silicone tube, the fabrication of the single-segment catheter is completed (j).

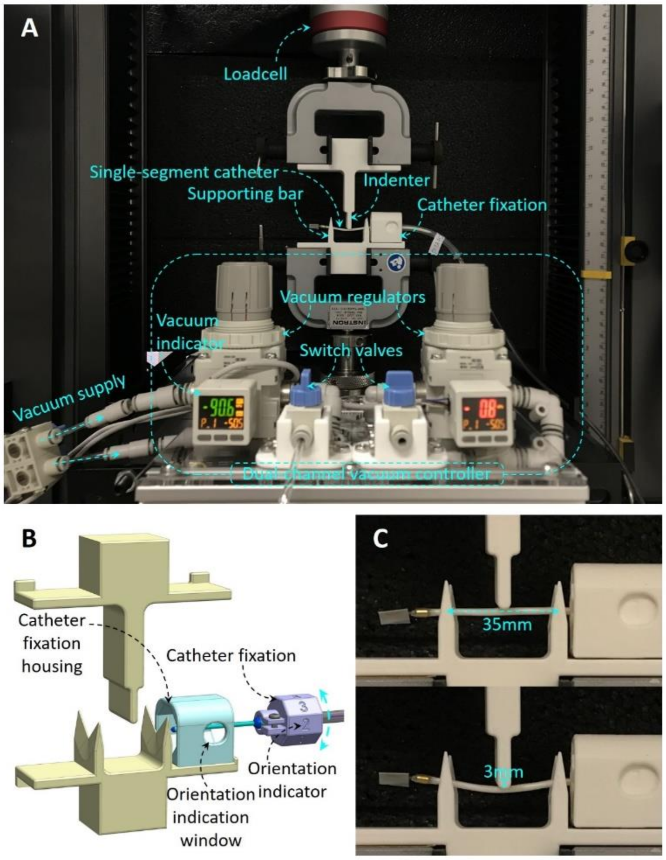

Fig. S7. Characterization setup for the single-segment catheter. (**A**) 3-point bending test setup fixed on the Instron machine along with the dual-channel vacuum controller. (**B**) Close-up view of the catheter fixation that allows bending tests at four orientations. After testing at one orientation, the catheter fixation will be drawn out, rotated by 45° to the next orientation, and then inserted in for testing. (**C**) 3-point bending test process with 3mm deflection.

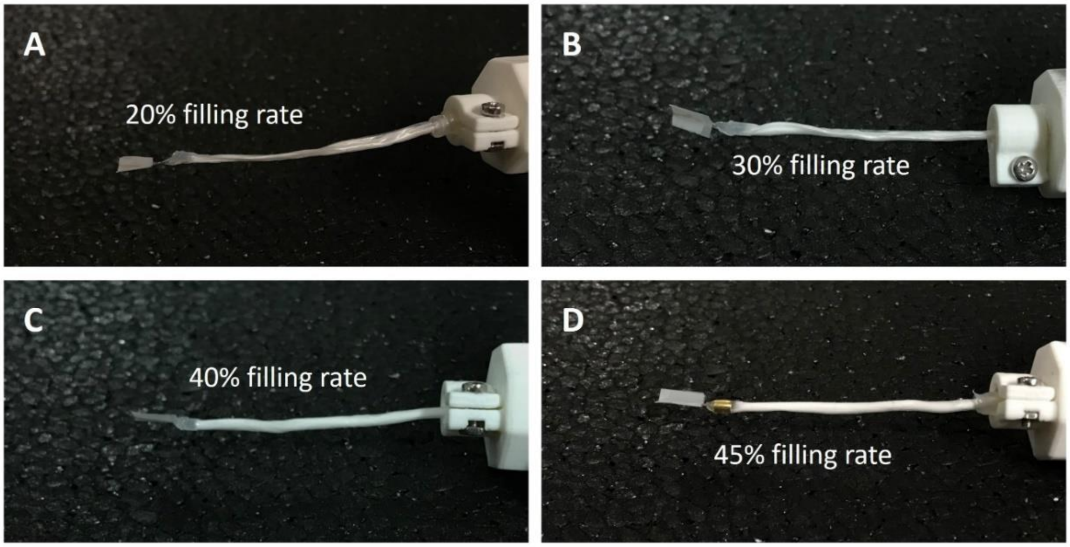

Fig. S8. Vacuumed states of the single-segment catheters at different filling rates. (**A**) 20%. (**B**) 30%. (**C**) 40%. (**D**) 45%.

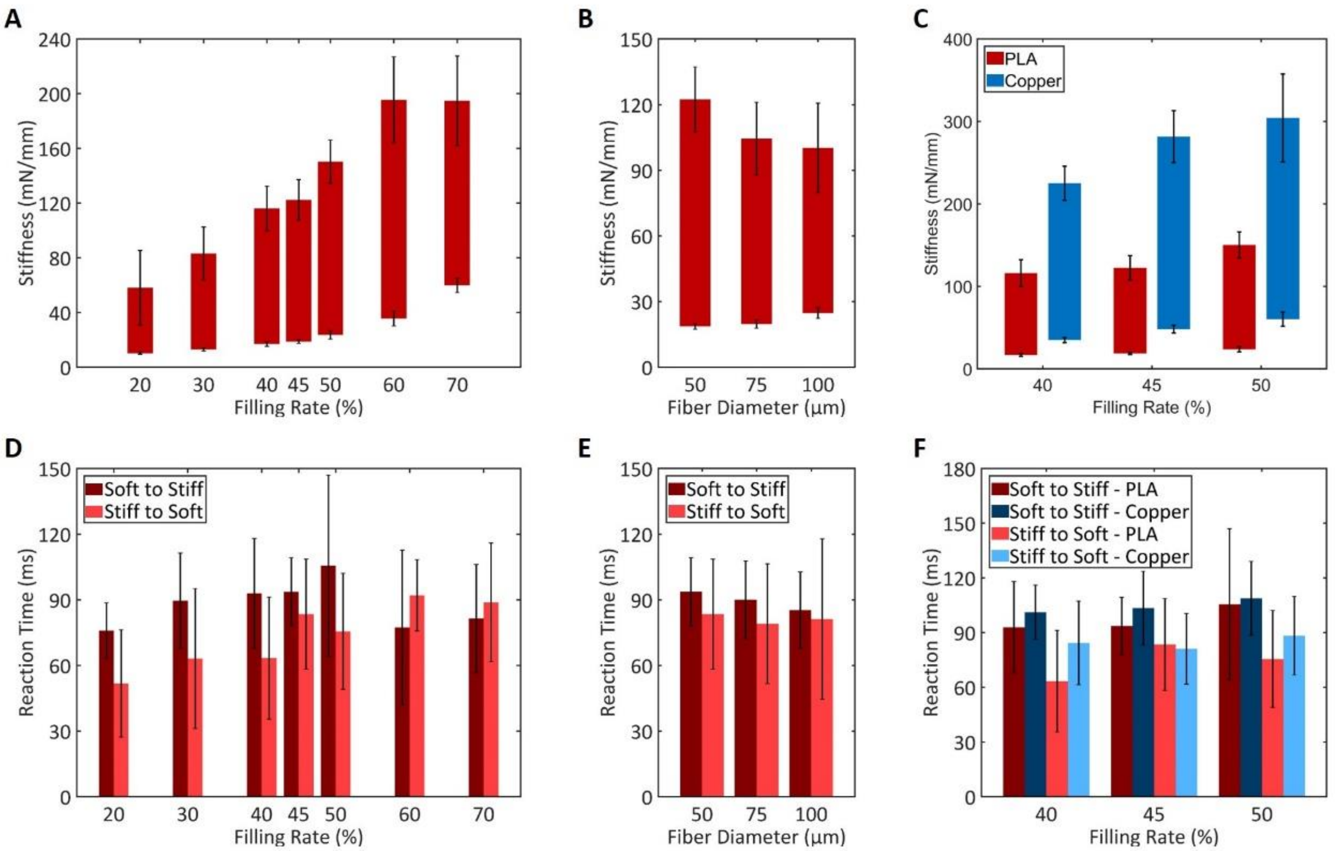

Fig. S9. Additional FJ characterization results. (**A**) Stiffness ranges of FJ with Ø50μm PLA fibers at different filling rates. (**B**) Stiffness ranges of FJ with PLA fibers of different diameters at 45% filling rate. (**C**) Stiffness ranges of FJ with Ø50μm PLA and copper fibers at the optimal filling rates. (**D**) FJ reaction times with Ø50μm PLA fibers at different filling rates. (**E**) FJ reaction times with PLA fibers of different diameters at 45% filling rate. (**F**) FJ reaction times with Ø50μm PLA and copper fibers at the optimal filling rates. Please note that all error bars represent one SD.

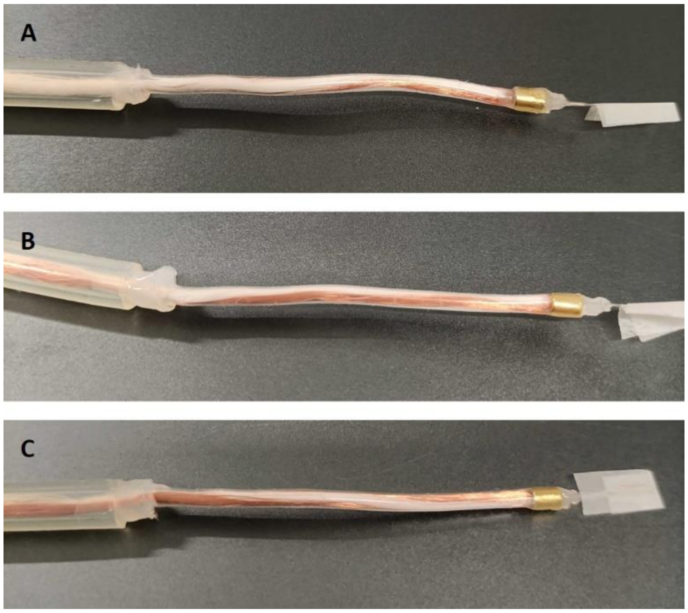

Fig. S10. Single-segment catheters with hybrid fiber bundles. (**A**) 75% PLA and 25% Copper. (**B**) 50% PLA and 50% Copper. (**C**) 25% PLA and 75% Copper.

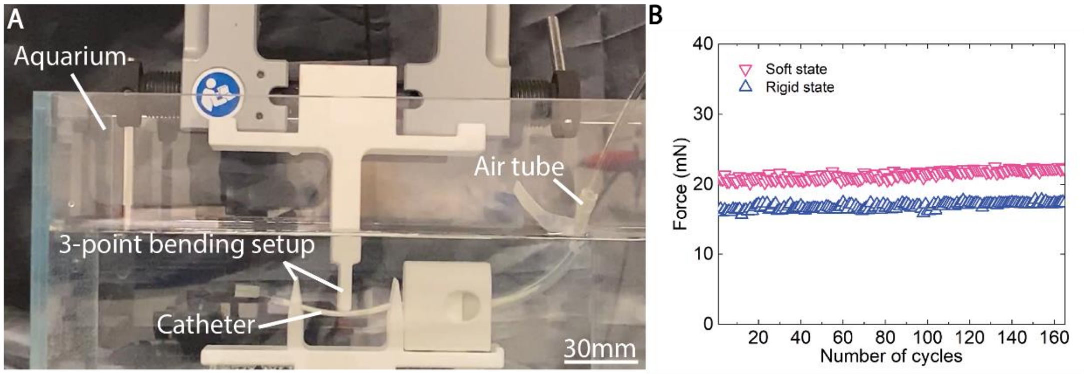

Fig. S11. Catheter robustness test. (**A**) Underwater test setup. (**B**) Force measurements up to 160 cycles.

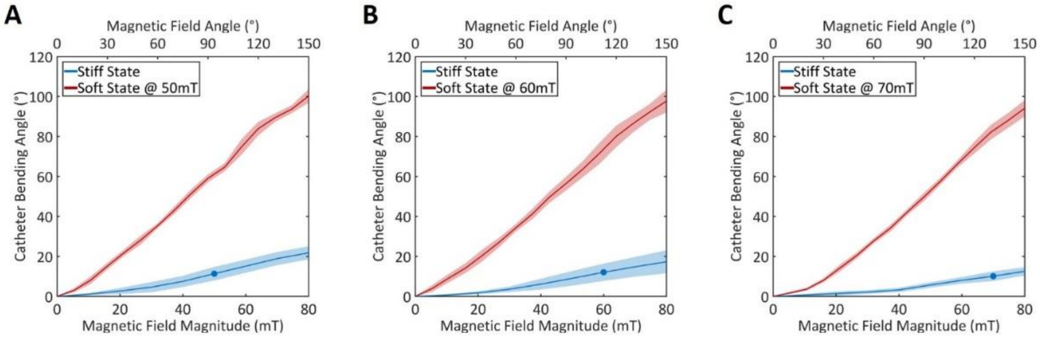

Fig. S12. Bending results of the catheters with hybrid fiber bundles under the RMN system. (**A**) 75% PLA – 25% copper. (**B**) 50% PLA – 50% copper. (**C**) 25% PLA – 75% copper. The blue dots highlight the small deflections at the corresponding MFDs for their tests in the stiff state.

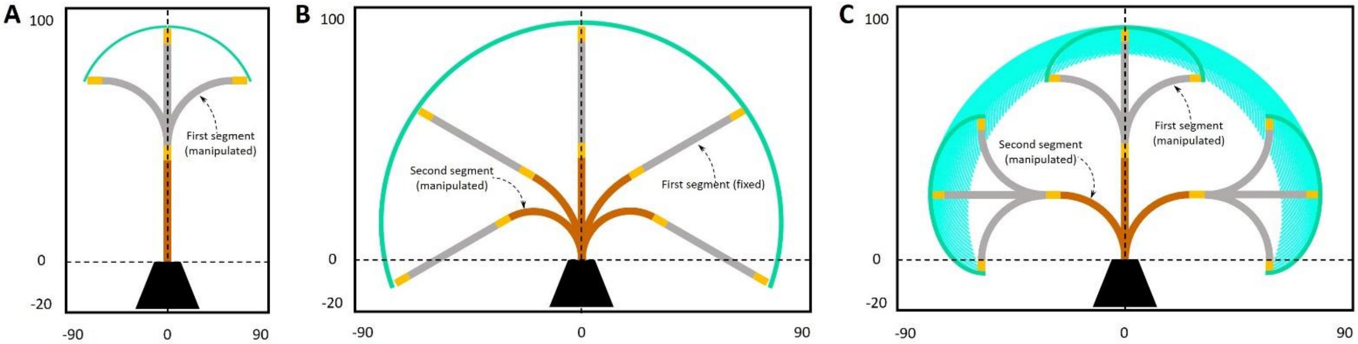

Fig. S13. The workspace of the two-segment FJ VS catheter. (**A**) The green arc represents the workspace of the tip of the first segment when the second segment is fixed, which corresponds to the typical workspace for single-segment catheters. (**B**) The green arc represents the workspace of the tip of the first segment (fixed) when the second segment is manipulated. In this configuration, the two magnets work together to bend the second segment, resulting in a larger bending range (-120° to 120°) (**C**) The green shade represents the workspace of the tip of the first segment when both segments are manipulated. Figure numbers are expressed in mm.

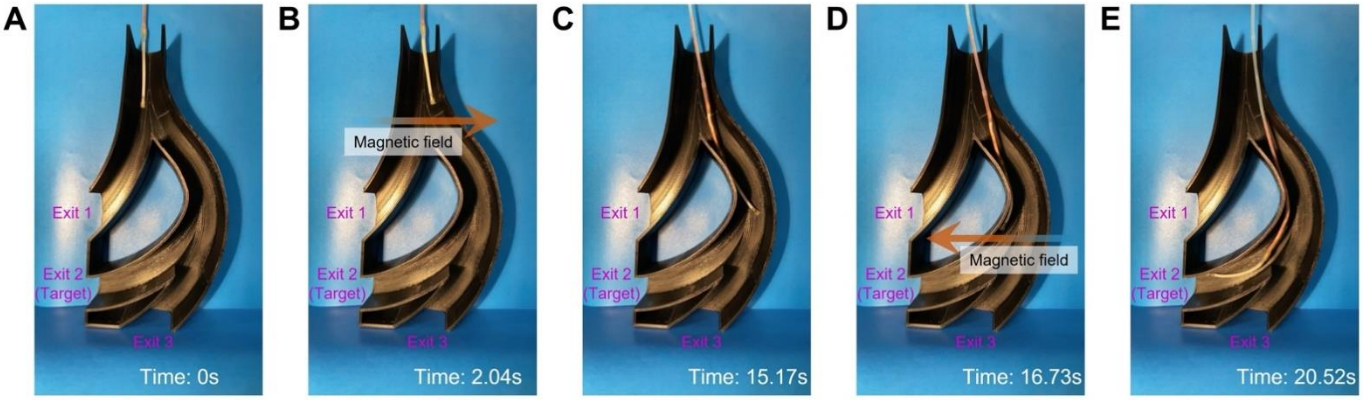

Fig. S14. Catheter navigating through a 2D maze. (**A**) Catheter (soft state) entering the maze. (**B**) Magnetic field navigating the catheter tip to the right path. (**C**) Catheter advancing. (**D**) Magnetic field navigating the catheter tip to the left path. (**E**) Catheter advancing and reaching the desired exit.

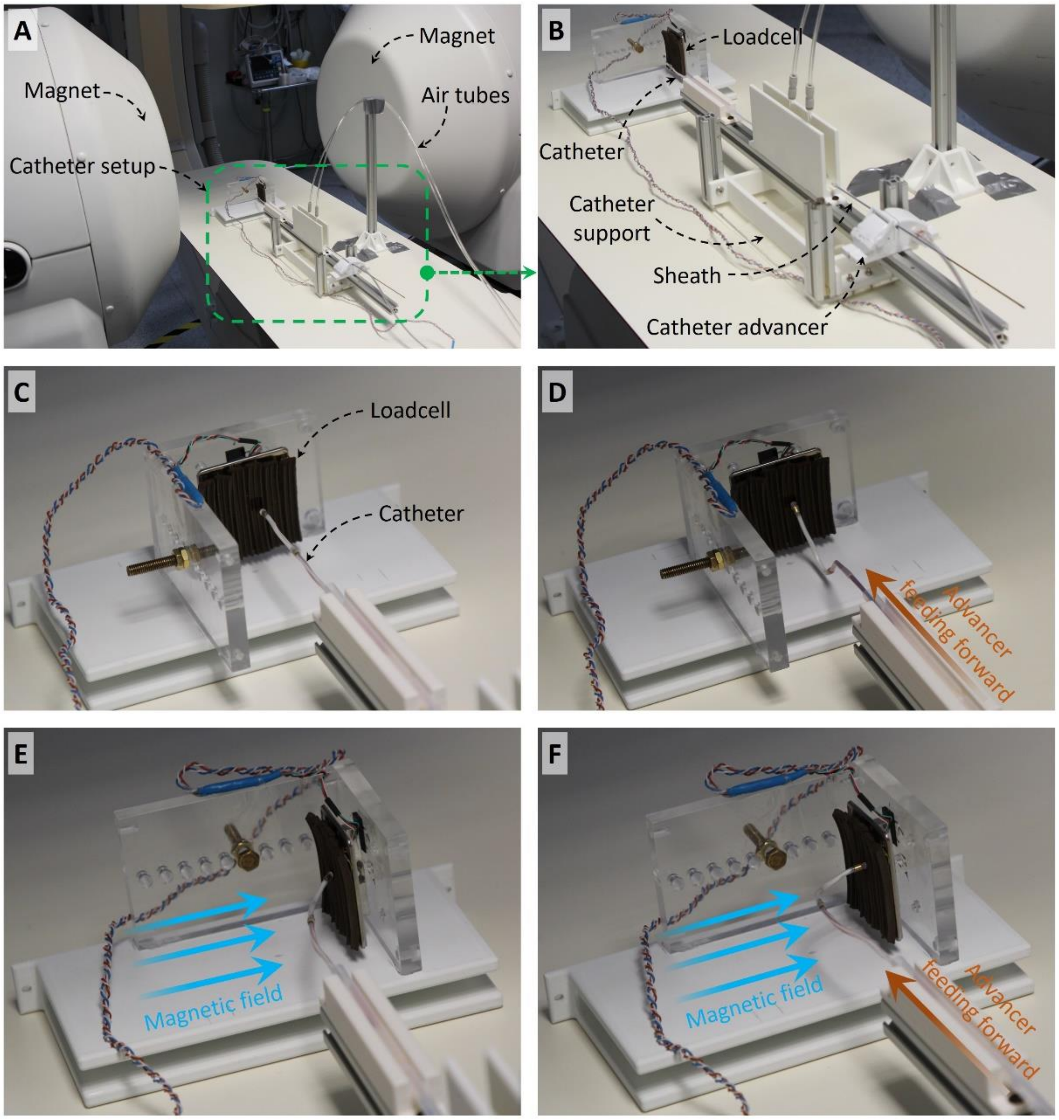

Fig. S15. Characterization of applied force in a clinical setting. (**A**) Catheter setup placed in the working area of the RMN system. (**B**) Close-up view of the catheter setup. (**C**) Perpendicular push onto the loadcell using a catheter advancer only. (**D**) Buckled catheter

after pushing. (**E**) Lateral touch with a load cell parallel to the catheter using only a magnetic field. (**F**) Lateral touch with a load cell using magnetic field and catheter advancer.

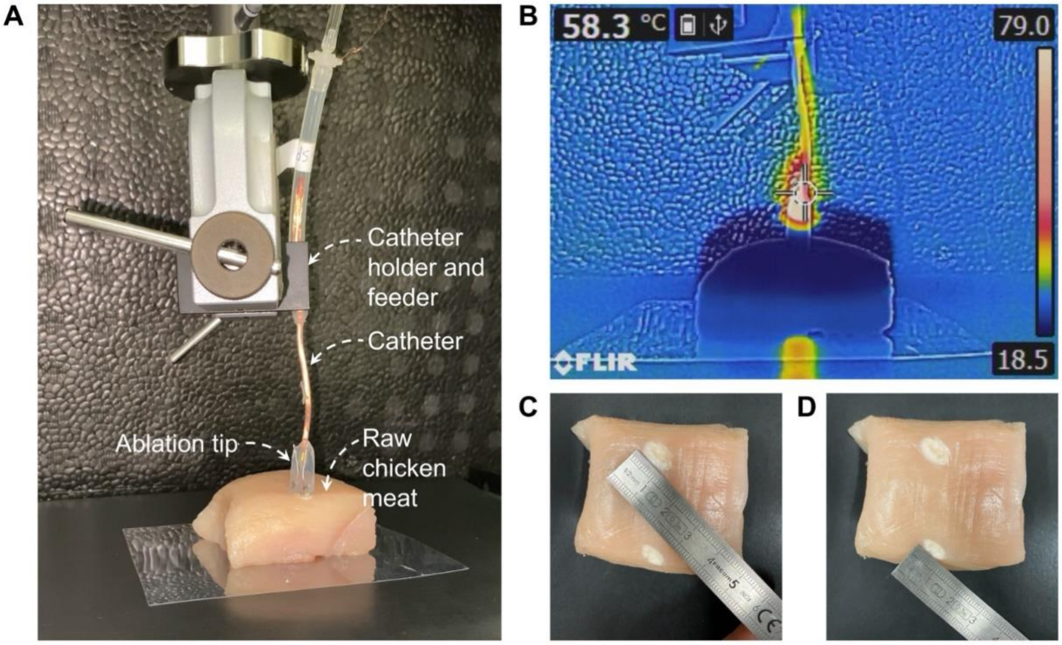

Fig. S16. ***Ex vivo*** **ablation using a VS FJ catheter.** (**A**) Ablation setup using a single-segment catheter with copper fibers. (**B**) Snapshot from a thermal camera showing tissue heating with the ablation tip of the catheter. (**C**) Lesion formed after ablation with the catheter in the rigid state. (**D**) Lesion formed after ablation with the catheter in the soft state.

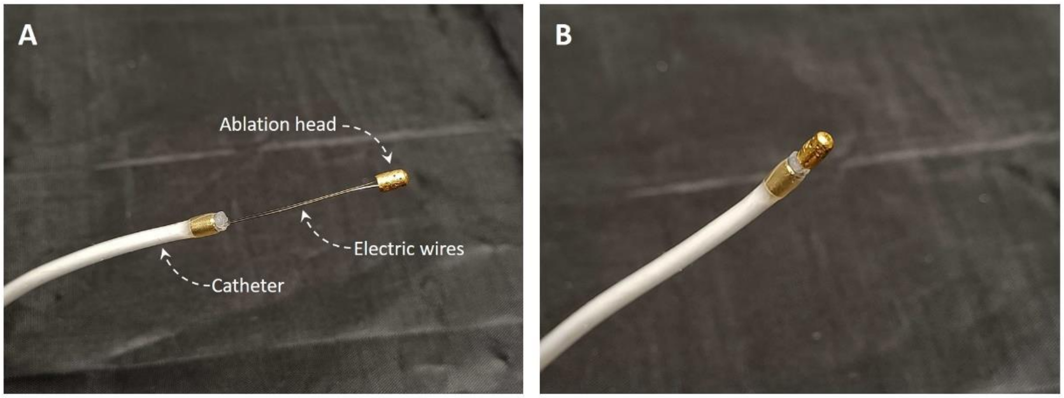

Fig. S17. Ablation tip integration to the catheter. (**A**) Electric wires of the ablation tip being inserted through the working channel. (**B**) Catheter with the integrated ablation tip integrated to the catheter. This is for demonstration purposes only, and all catheter experiments are conducted without the ablation tip. Future integration of an ablation tip will require the insertion of multiple Ø50µm copper wires (instead of the thick wires used in current commercial tips) to minimize stiffness effects while meeting power requirements. Please note that we were not able to use this ablation tip (disassembled from a commercial catheter) for the ablation test due to the lack of its user manual.

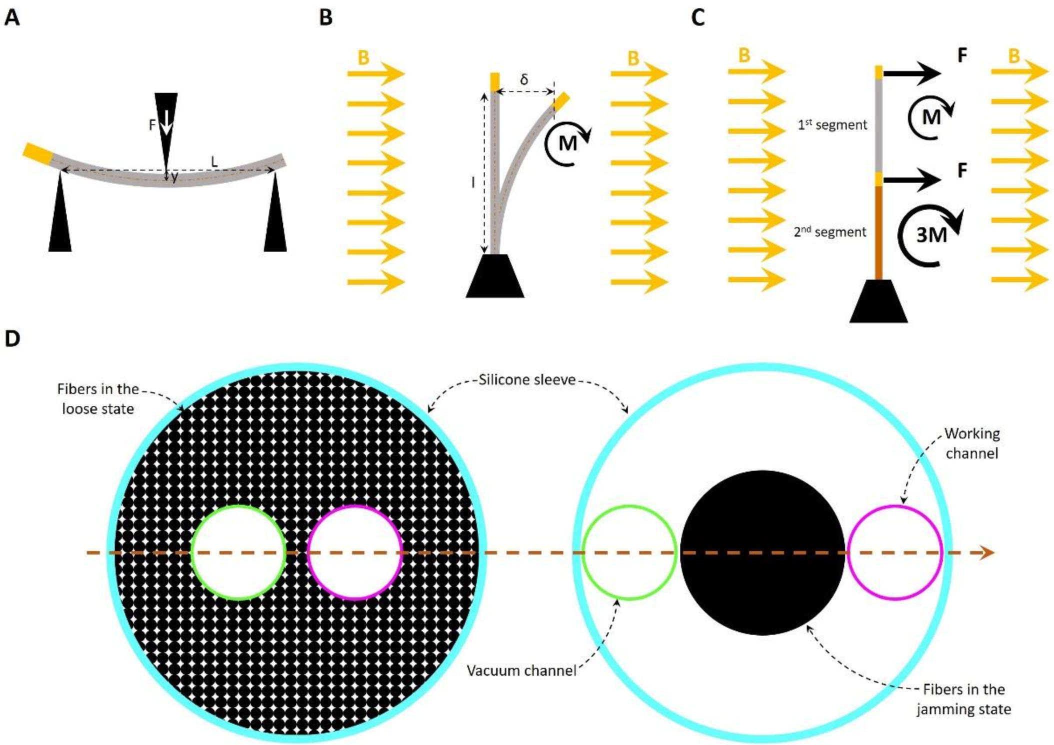

Fig. S18. Different loading scenarios of the catheters. (**A**) The single-segment catheter in the 3-point bending test. (**B**) The single-segment catheter in a magnetic field. (**C**) The two-segment catheter in a magnetic field. (**D**) Simplified cross-section view of the catheter with different components in soft (left) and stiff (right) states.

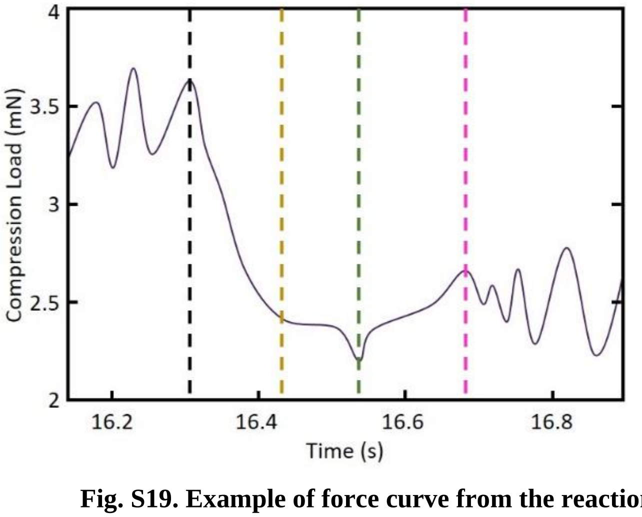

Fig. S19. Example of force curve from the reaction time experiment. The vertical dashed lines are time mark lines for the last peak in the high force level (black), the start of the low force level (rough estimation) (yellow), the first trough of the low force level (green) and the first peak of the low force level (pink). The grey horizontal dashed lines are the two force levels.

Table S1. Summary of existing variable stiffness catheters in stiffness change time.

Existing variable stiffness catheters			Time (s)		
Article title	Phase-changing material	Year	Softening	Stiffening	Full cycle
Magnetic continuum device with variable stiffness for minimally invasive surgery (11)	Low-melting-point alloy	2020	15	80	95
A submillimeter continuous variable stiffness catheter for compliance control (12)	Low-melting-point alloy	2021	10	60	70
Thermoset shape memory polymer variable stiffness 4d robotic catheters (13)	Thermoset shape memory polymer	2022	9	60	69
A variable stiffness magnetic catheter made of a conductive phase-change polymer for minimally invasive surgery (14)	Conductive shape memory polymer	2022	36	90	126
				Average	90

Table S2. Information about the materials used in the catheter and the fabrication.

	Items	Material	Supplier	Outer diameter (mm)	Inner diameter (mm)	Wall thickness (mm)	Length (mm)	Elastic modulus (MPa)
Main components	1st Silicone sleeve<br>2nd Silicone sleeve	DragonSkin0020	Smooth-On, Inc.	2.3	2	0.15	50<br>60	0.34
	Silicone glue	Sil-poxy	Smooth-On, Inc.	NA	NA	NA	NA	2.7
	Working channel<br>Vacuum channel	Polytetrafluoroethylene (PTFE)	Zeus, Inc.	0.5842	0.4826	0.0508	450	400
	PTFE tube	PTFE	Zeus, Inc.	2.3114	2.0828	0.1143	200	400
	PLA fiber	Polylactide Acid (PLA)	Ultimaker B.V.	0.05	NA	NA	40	2763
	Supporting CF rod	Carbon fiber (CF)	Suter Kunststoffe AG	0.3	NA	NA	15	228000
	Copper fiber	Copper	Xinye Electric, Inc.	0.05	NA	NA	80	117000
	Silicone tube	Silicone	ROTIMA AG	4	2	1	30	4.3
	PU tube	Polyurethane (PU)	SMC. Ltd	4	2	1	30 & 70	2580
	Magnet	Neodymium+Iron+Boron (NdFeB)	HKCM Engineering e.K.	2.5	1.25	0.625<br>Dipole moment:	3.75<br>0.01187 Am^2	NA
	Barb fitting	Polypropylene (PP)	Foreshine. Ltd	Max diameter of two ends		2.4 mm	3.9 mm	NA
	Cross fitting	Polybutylene terephthalate (PBT)	SMC. Ltd	Port size		4 mm		NA
Assistive components	Assistive tube	PTFE	Zeus, Inc.	2.1	2	0.0508	70	400
	Assistive CF rod	Carbon fiber	Suter Kunststoffe AG	0.3	NA	NA	500	228000

Table S3. Stiffness and SCF at initial and medium deflection range for catheter scale FJ at varied vacuum levels.

FJ spec:	Ø50μm PLA fiber, 45% filling rate	0 kPa (soft state)	Vacuum -25kPa	Vacuum -50kPa	Vacuum -75kPa	Vacuum -95kPa
0 - 0.2 mm deflection	Stiffness (mN/mm)	18.54	90.26	139.58	173.15	226.31
	SCF		4.87	7.53	9.34	12.21
0.2 - 0.8 mm deflection	Stiffness (mN/mm)	18.54	39.17	60.57	78.12	100.86
	SCF		2.11	3.27	4.21	5.44

Table S4. Calculation based on the hybrid fibre bundle characterization results.

Fiber composition	Stiffness state	Stiffness (N/m)	Flexural rigidity *EI* (×10-6 N·m2)	MFD (mT) needed to achieve a deflection of 5mm	15mm	20mm	Recommended MFDs (mT) for the soft states
100% PLA	soft	18.67	16.67	8.78	26.34	35.12	30*
0% copper	stiff	122.33	109.27	57.54	172.61	230.14	
75% PLA	soft	29.75	26.57	14	41.98	55.97	50
25% copper	stiff	154.25	137.78	72.55	217.64	290.19	
50% PLA	soft	36.58	32.68	17.21	51.62	68.82	60
50% copper	stiff	184.42	164.73	86.73	260.2	346.94	
25% PLA	soft	46.86	41.86	22.04	66.12	88.16	70
75% copper	stiff	232.64	207.8	109.41	328.24	437.66	
0% PLA	soft	48.08	42.95	22.61	67.84	90.46	80
100% copper	stiff	281.56	251.49	132.42	397.26	529.68	

* Although 30mT is recommended, 40mT is used for the testing of the catheter with 100% PLA fiber for larger deflections. Please refer to section S4 for a detailed explanation.

Table S5. Relation between spool spinning speed and fiber thickness.

Spool spinning velocity (rad/s)	Spool edge linear velocity (mm/s)	Resultant fiber thickness (μm)
12.5π	1963.5	46.6
10π	1570.8	50.1
6.25π	981.75	60.8
3.968π	623.33	74.8
3.125π	490.875	85.5
2.294π	360.275	100.1
2.084π	327.25	104.4
1.5625π	245.44	115.1

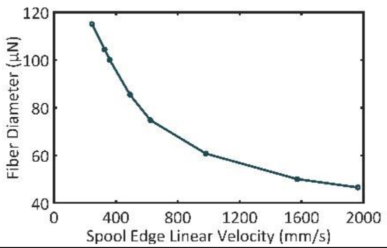

Table S6. Comparison between experimental and calculated stiffness of the catheters.

State	Method	Stiffness (mN/mm)
Soft	Calculated stiffness (Lower limit)	3.64
	Experimental stiffness measured within 1mm deflection	18.54
Stiff	Experimental stiffness measured within 1mm deflection	121.3
	Experimental stiffness measured within 0.2mm deflection	226.3
	Calculated stiffness (Upper limit)	494.9

Movie S1. Fabrication of two-segment catheter.
Movie S2. Single-segment catheter characterization.
Movie S3. Demonstration of fast stiffness change of catheter.
Movie S4. Two-segment catheter bending demonstration.
Movie S5. Two-segment catheter demonstration with a 3D phantom of a human heart.
Movie S6. Two-segment catheter navigating through a 2D maze.
Movie S7. Catheter ablation test.
